# The Potential Role of Genetics, Environmental Factors, and Gut Dysbiosis in the Aberrant Non-Coding RNA Expression to Mediate Inflammation and Osteoclastogenic/Osteogenic Differentiation in Ankylosing Spondylitis

**DOI:** 10.3389/fcell.2021.748063

**Published:** 2022-01-20

**Authors:** Hsien-Tzung Liao, Chang-Youh Tsai, Chien-Chih Lai, Song-Chou Hsieh, Yi-Syuan Sun, Ko-Jen Li, Chieh-Yu Shen, Cheng-Han Wu, Cheng-Hsun Lu, Yu-Min Kuo, Tzu-Hao Li, Chung-Tei Chou, Chia-Li Yu

**Affiliations:** ^1^ Division of Allergy, Immunology and Rheumatology, Taipei Veterans General Hospital, National Yang-Ming Chiao-Tung University, Taipei, Taiwan; ^2^ Division of Rheumatology, Immunology and Allergy, Department of Internal Medicine, National Taiwan University Hospital and National Taiwan University College of Medicine, Taipei, Taiwan; ^3^ Division of Allergy, Immunology and Rheumatology, Taipei, Taiwan; ^4^ Shin Kong Wu Ho-Su Memorial Hospital, Taipei, Taiwan

**Keywords:** ankylosing spondylitis, HLA-B27, endoplasmic reticulum aminopeptidase, gut dysbiosis, IL-23/IL-17 axis, enthesitis, circular RNA, osteogenesis/osteoclastogenesis

## Abstract

Ankylosing spondylitis (AS) or radiographic axial spondyloarthritis is a chronic immune-mediated rheumatic disorder characterized by the inflammation in the axial skeleton, peripheral joints, and soft tissues (enthesis, fascia, and ligament). In addition, the extra-skeletal complications including anterior uveitis, interstitial lung diseases and aortitis are found. The pathogenesis of AS implicates an intricate interaction among HLA (HLA-B27) and non-HLA loci [endoplasmic reticulum aminopeptidase 1 (*ERAP1*), and interleukin-23 receptor (*IL23R*), gut dysbiosis, immune plasticity, and numerous environmental factors (infections, heavy metals, stress, cigarette smoking, etc.) The latter multiple non-genetic factors may exert a powerful stress on epigenetic regulations. These epigenetic regulations of gene expression contain DNA methylation/demethylation, histone modifications and aberrant non-coding RNAs (ncRNAs) expression, leading to inflammation and immune dysfunctions. In the present review, we shall discuss these contributory factors that are involved in AS pathogenesis, especially the aberrant ncRNA expression and its effects on the proinflammatory cytokine productions (TNF-α, IL-17 and IL-23), T cell skewing to Th1/Th17, and osteoclastogenic/osteogenic differentiation. Finally, some potential investigatory approaches are raised for solving the puzzles in AS pathogenesis.

## Introduction

**FIGURE 1:**
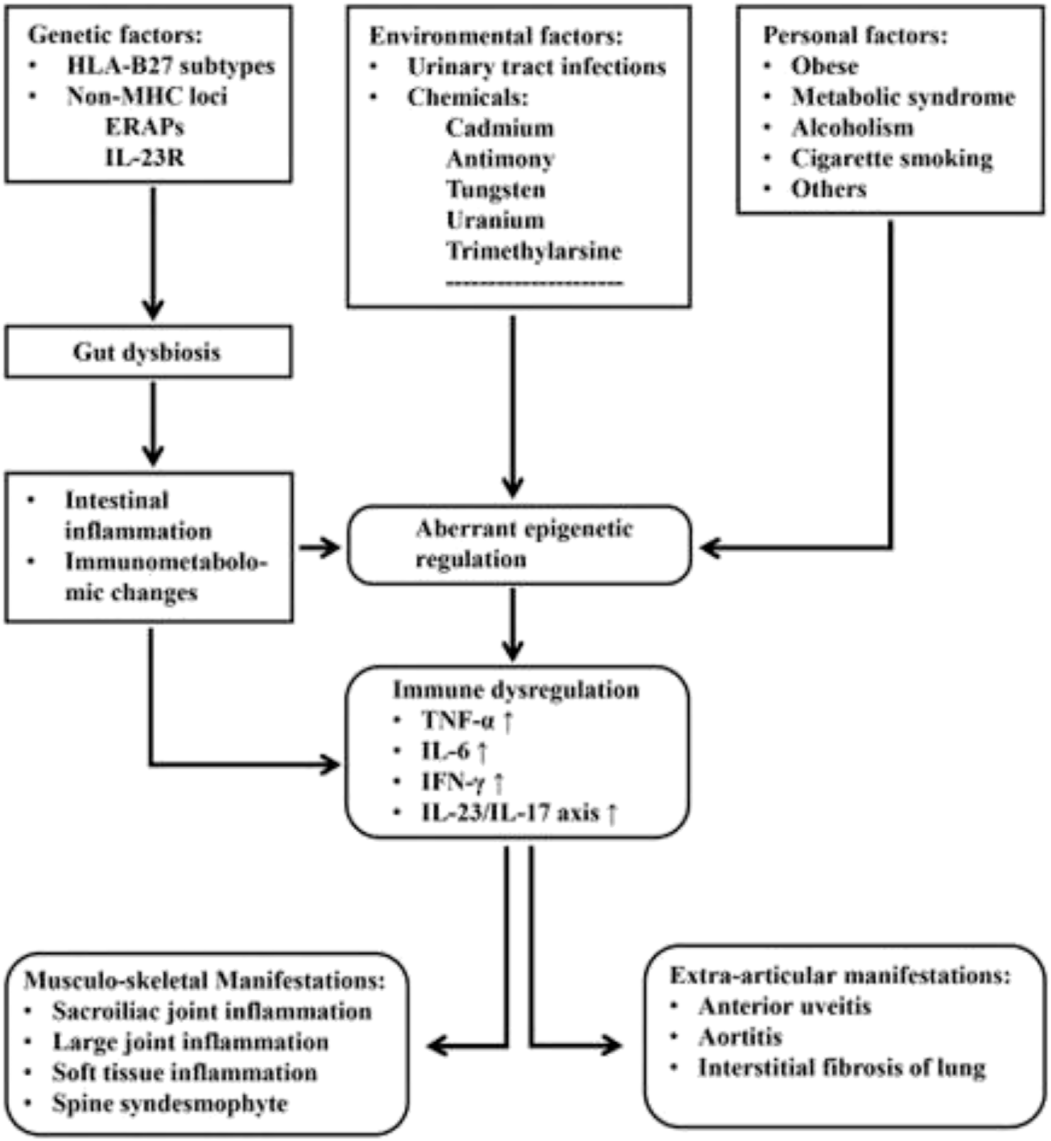
A model depicting the possible pathogenetic elements in patients with ankylosing spondylitis: Many genetic, environmental and personal factors are involved in the induction of gut dysbiosis and aberrant epigenetic regulation. Subsequently, these induced pathological changes may lead to intestinal inflammation, immunometabolomic alterations and immune dysfunction. Finally, the musculo-skeletal and extra-articular manifestations ensue.

Ankylosing spondylitis (AS) and allied diseases, also known as radiographic axial spondyloarthritis (r-AxSpA), are a set of common immune-mediated inflammatory arthritides mainly affecting axial skeleton, particularly the sacroiliac joints, non-synovial spinal joints and enthesis (the connection between tendon and bone). Chronic pain with ankylosis of the spine and disability are the characteristics of AS/r-AxSpA. It is estimated that 0.5% of the world population are affected by AS, rendering it an important health-care and socioeconomic issue. Although the exact etiology and pathogenesis of AS/r-AxSpA remain obscure, genetic predisposition by human leukocyte antigen (HLA)-B27 subtypes is known to have a strong association with the disease. However, other genetic loci of non-major histocompatibility complex (MHC) including endoplasmic reticulum aminopeptidase (*ERAP1*) and interleukin 23 receptor (*IL-23R*), gut microbiome, local immune-metabolomics in gastrointestinal (GI) tract and joints, as well as T cell plasticity may also be implicated in its pathogenesis (Voruganti and Bowness, 2020; Hwang et al., 2021). In addition to musculoskeletal manifestations, the extra-articular complications such as anterior uveitis (AU), interstitial pulmonary fibrosis, osteoporosis, syndesmophyte formation, and cardiovascular diseases (e.g., aortitis) may also occur (El Maghraoui, 2011; Stolwijk et al., 2015; Redeker et al., 2020). On the other hand, the effects of some environmental factors have been recognized through studying the relationships of different urinary tract infections and the contaminating inorganic compounds in the urine of AS patients. Shiue (2015) have reported significantly higher urine concentrations of cadmium, antimony, tungsten, uranium, and trimethylarsine in patients with AS. In a prospective cohort study, Zeboulon-Ktorza et al. (2013) have demonstrated a moderate statistical significance between vaccination and an elevation of Bath ankylosing spondylitis disease activity index (BASDAI) in AS. In spite of these factors, the investigations on the prevalence and influence of multi-morbidities in the disease activity of patients with AS revealed no direct cause-effect relationship between environmental factors and aberrant epigenetic regulation. Fitzgerald et al. (2020) have found that only the individual factors of metabolic syndrome are associated with more severe disease. Based on these backgrounds, we’ll discuss consecutively the possible molecular bases underlying the development of AS from viewpoints of genetics, gut dysbiosis, aberrant epigenetic regulation, and immune dysregulation to further understand the immunopathogenesis of AS. A proposed multifactorial co-morbid model in causing AS is depicted in Figure 1.

## Proposed Molecular Pathogenetic Mechanisms for AS

At least five hypotheses to account for the molecular pathogenesis in AS patients have been proposed: 1) arthritogenic peptide stimulation, 2) unfolding protein response, 3) HLA-B*27 homodimer formation, 4) dysfunction of endoplasmic reticulum aminopeptidase (ERAP), and 5) gut inflammation caused by microbiota dysbiosis (Sharip and Kunz, 2020). We’ll first discuss in detail the link of genetics (HLA subtypes and non-HLA loci) with these hypotheses in the next section.

## Implication of Genetics in the AS Pathogenesis

**FIGURE 2:**
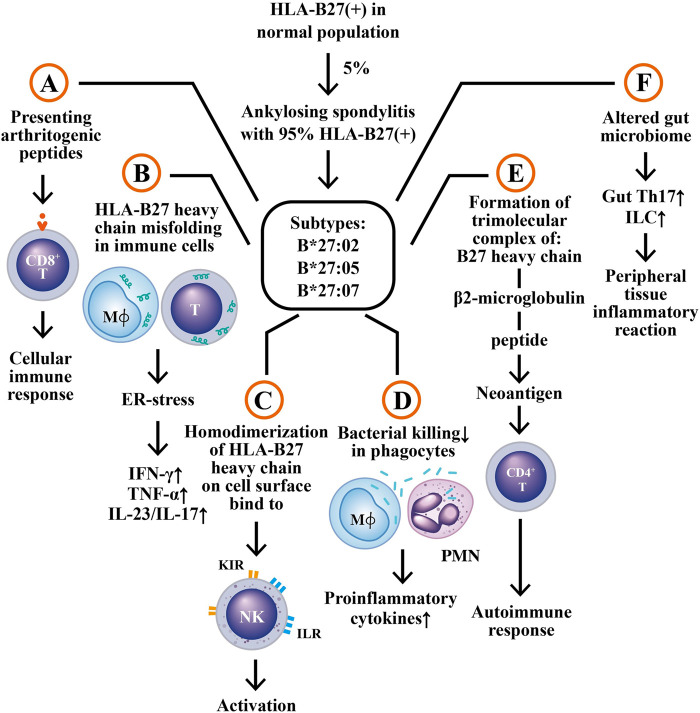
The six potential mechanisms by which HLA-B27 subtypes may be involved in AS pathogenesis. **(A)** The presentation of arthritogenic peptides from endogenous or intestinal microbes to HLA-B27-expressing CD_8_^+^ T cells to elicit cellular immune responses; **(B)** Misfolding of HLA-B27 subtype heavy chain in macrophages and T cells to induce endoplasmic reticulum stress (ER stress) and subsequent production of immune- and inflammation-related cytokines; **(C)** Homodimerization of HLA-B27 heavy chains, binding to surface KIR and ILR molecules to activate NK cells; **(D)** Defective bacterial killing activity of the HLA-B27 (+) phagocytes to enhance pro-inflammatory cytokine productions; **(E)** Formation of a trimolecular complex of “B27 heavy chain + β2 microglobulin + peptide” as neoantigen to stimulate autoimmune responses of CD4^+^ T cell; **(F)** Altered gut microbiome in HLA-B27 (+) patients to enhance the generation of Th17 and innate like T cells (ILC) in the alimentary tract, which then migrate to peripheral tissues to induce inflammatory reactions. KIR, kill cell immunoglobulin like receptor; ILR, immunoglobulin like receptor; IFN, interferon; TNF, tumor necrosis factor; IL, interleukin. M*ϕ*, macrophage.

HLA-B27 positivity is present in 85–95% of patients with AS/r-AxSpA in different ethnicities in the world (Jamalyaria et al., 2017; Reveille, et al., 2019). However, only 5% of HLA-B27 (+) individuals in the general population have AS/r-AxSpA or undifferentiated spondyloarthropathy (USpA) (Akkoc and Khan, 2005). Nevertheless, HLA-B27 is still considered to be an important genetic factor highly associated with the development of AS. Six mechanisms have been suggested for the disease association; (A) The presentation of an arthritogenic peptide (Faham et al., 2017) to CD8^+^ T lymphocyte enriched in the inflamed joint (Gracey et al., 2020); (B) The presence of subtype HLA-B27 heavy chain, B*27:02, with a greater tendency to fold erroneously and accumulate in endoplasmic reticulum (ER)-derived vesicles. This may lead to a response to an unfolded peptide that can activate intracellular biochemical events and upregulate proinflammatory cytokines such as interferon-γ (IFN-γ), IL-17 and IL-23 (Jeanty et al., 2014; Jah et al., 2020; Navid et al., 2021); (C) A striking tendency of HLA-B27 heavy chains to adhere with each other, forming homodimers. These homodimers on the cell surface can be recognized by killer cell immunoglobulin-like receptor (KIR) and leukocyte immunoglobulin-like receptor (LILR) on natural killer (NK) cells (Lim Kam Sian et al., 2019); (D) HLA-B27 bearing individuals show impaired intracellular killing of pathogenic microorganisms that can lead to the persistence of intracellular bacterial pathogens and consequently stimulating proinflammatory cytokine production (Sahlberg et al., 2012); (E) Trimolecular complex (B27 heavy chain, α2 microglobulin and peptide) of HLA-B27 itself, free heavy chain, or homodimers of HLA-B27 may be recognized as neoantigens by the T cell receptor on CD_4_^+^ T lymphocytes in activating autoimmune responses (Boyle et al., 2001), (F) HLA-B27 bearing individuals generate an altered intestinal microbiome to increase HLA-B27 subtype expression involved in the immunopathogenesis of AS. These six potential effects conferred by HLA-27 subtypes are illustrated in Figure 2.

In addition to the HLA-B27 subtypes, genome-wide association studies (GWAS) have also identified more than 100 non-HLA-B27 loci associated with AS. These loci include machinery for antigen presentation (*ERAP*1 and *ERAP*2), some loci in Th17 cells (encoding *IL-6R*, *IL-23R*, *TYK1* and *STAT3*) and others in macrophages and T cells (encoding *IL-7R*, *CSF2*, *RUNX3* and *GPR65*) (Hanson and Brown, 2017; Kavadichanda et al., 2021; Wordsworth et al., 2021).

While over 90% of AS patients have an HLA-B*27 haplotype, only around 5% of individuals bearing HLA-B*27 develop AS. This implies a presence of additional risk factors to facilitate the disease development. It has been reported that strong epistatic gene-gene interactions between *HLA-B27* and specific *ERAP1* variants may work (Reveille, 2012). In addition, one of the major functions of ERAP1 is to trim endogenous peptides before their binding onto MHC-class I molecules. The epistatic interactions between *ERAP1* and *HLA* allele may be involved in AS pathogenesis (Evans et al., 2011; Cortes et al., 2015). Furthermore, some reports have unveiled that ERAP1 can suppress both innate and adaptive immune responses (Goto et al., 2011; Aldhaman et al., 2013; Aldhamen et al., 2015). In experiments to evaluate bone morphogenesis of the axial skeletons in *ERAP1* −/− mice, the authors have found that *ERAP1* −/− mice can serve as a useful model for AS to observe spinal ankyloses, osteoporosis and inflammation. In addition, it can reduce both Tγ1-like regulatory T cells and tolerogenic dendritic cells (Pepelyayeva et al., 2018), which are important for Tγ1 differentiation and function (Wakkach et al., 2003). Finally, inflammation in the spine happens. The two ERAP genes, *ERAP1* and *ERAP2*, are ubiquitous, zinc-dependent, and multifunctional, playing a role in several HLA-class I-mediated diseases in addition to AS (McGonagle et al., 2015; de Castro et al., 2016; Vitulano et al., 2017; Hansen et al., 2018). However, it is quite interesting that only functional polymorphisms of *ERAP1* affect AS risk in HLA-B27 bearing individuals (Evans et al., 2011). *ERAP2* appears independent from HLA-B27 (Robinson et al., 2015). Recently, authors discovered that a single nucleotide polymorphism (SNP), *rs75862629*, in the *ERAP2* promoter region can influence the *ERAP2* expression that can be counteracted by a higher expression of *ERAP1* (Paladini et al., 2018). Furthermore, this SNP was found capable of modulating simultaneously the expression of both *ERAP1* an *ERAP2* and protecting hosts from AS in HLA-B27-positive individuals in Sardinia populations (Paladini et al., 2019). In contrast, a significant association between *ERAP1* polymorphisms, *rs30187* and *rs27037*, conferred an increased risk for AS in East Asian population (Jiang et al., 2018). A meta-analysis of *ERAP1* gene polymorphism unveiled that SNPs *rs27044* and *rs30187* are significantly associated with AS susceptibility in Caucasians rather than Asians (Gao et al., 2020). Silencing of *ERAP1* suppresses HLA-B27-free heavy chain expression and Th17 responses in AS (Chen L. et al., 2016; Nakamura et al., 2021). Moreover, the effects of *ERAP1* polymorphisms on the proinflammatory and anti-inflammatory cytokine expressions in AS patients have also been investigated. It was found that T allele of *rs30187* and C allele of *rs2287987* were associated with risk of HLA-B27-positive AS development by significant overexpression of proinflammatory cytokines (IL-17A, IL-17F, IL-23, TNF-α and IFN-γ) and under-expression of anti-inflammatory cytokines (IL-10 and TGF-β) in PBMC of HLA-B27 (+) AS patients (Babaie et al., 2020)

On the other hand, the studies exploring the association between *TNF* polymorphisms and AS remain inconclusive. A meta-analysis has revealed that the A allele in *TNF*-238 and *TNF*-308, the C allele in *TNF*-1031, the T-allele in *TNF*-850 and *rs769138* are significantly associated with AS susceptibility in the total population (Hu et al., 2021). In addition, *IL-12B* gene polymorphism (Ivanova et al., 2019), *IL-23R* SNPs and *IL-10*-819 polymorphism (Xia et al., 2018) are associated with AS pathogenesis.

In short summary, the aminopeptidases, ERAP1 and ERAP2, trim the peptides to a length suitable for fitting into the groove of MHC class I molecules for protection from viral infection. The epistatic interactions between HLA-B27 peptide repertoires can determine the innate immunological function of HLA-B27 such as antigen presentation to T cells. However, the process also produces a by-product, an intracellular misfolded HLA-B27, or an HLA-B27 homodimer on the cell surface, which can elicit ER stress responses, autophagic engulfment, or innate immune responses. These aberrant interplays between HLA-B27 and ERAP1/ERAP2 result in a deviation of the physiological function from defending against infections to pathological induction of spondyloarthritis (Vitulano et al., 2017). Moreover, the accumulation of misfolded HLA-B27 heavy chain along with β2-microglobulin and ER chaperones (calnexin, calreticululin, BiP, 94kD glucose-regulated protein) into ER-derived vesicles is different from the peptide-loading complex. These abnormal behaviors may become the unique features of HLA-B27 subtypes predisposing to AS (Jah et al., 2020).

## Roles of Hazard HLA Allele-Associated Gut Dysbiosis and Its Metabolites in the Pathogenesis of AS

Sufficient evidence has demonstrated the pathogenic roles of gut microbiome in inflammatory arthritides including SpA. Human gut is colonized with bacteria, viruses, and fungi, which actively interact with each others (Li M. et al., 2019). More than 100 trillion bacteria reside in the mammalian gut to establish a symbiotic relationship. This intimate association can influence many aspects of the host’s metabolism, physiology and immunity. Accordingly, intestinal dysbiosis may play an important role in the development of AS by altering intestinal permeability, stimulating immune responses, and exerting molecular mimicry (Yang et al., 2016).

To determine whether AS patients’ guts carry a distinct microbial signature from that in the healthy individuals, 16S ribosomal RNA sequences of microbiome in the terminal ileum were analyzed. A higher abundance of five families of bacteria (*Lachnospiraceae*, *Rumincoccaceae*, *Rikenellaceae*, *Porphyromonadaceae*, and *Bacteroidaceae*) were reported (Costello et al., 2015). In addition, a quantitative metagenomic study based on the shotgun sequencing of the gut microbial DNA in Chinese AS patients has revealed the increases in *Prevotella melaninogenica*, *Prevotella copei*, and *Prevotella* spp. C561, but a decrease in *Bacteroides* spp. In addition, *Bifidobacterium* genus, which is commonly used in probiotics, was found accumulated in AS patients (Wen et al., 2017). An amplicon gene sequencing study on 16S ribosomal RNA disclosed the genus *Dializer* as a microbial marker for disease activity in SpA (Tito et al., 2017). Recently, by using real-time polymerase chain reaction (PCR) to analyze intestinal microbiota in stool samples, investigators have found that the total quantity of bacteria is decreased in patients with AS. The intestinal dysbiosis is associated with a more severe articular disease as evidenced by the findings that *Bifidobacterium* and *Lactobacillus* were increased in active AS patients (Cardoneanu et al., 2021). Besides, gut microbiota and mycobiota in AS patients were detected by 16S rRNA gene and ITS2-based DNA sequencing. It revealed that *Proteobacteria* was increased and *Bacteroides* was decreased. This abnormality was resulted from enrichment of *Escherichia*-*Shigella*, *Veillonella*, *Lachnospiraceae* NK4A136 group, and reduction of *Prevotella* strain 9, *Megamona*, and *Fusobacterium*. On the other hand, the mycobiota in alimentary tract of AS patients, as shown by 16S rRNA gene analyses, exhibited higher expression of *Ascomycota* (especially the class of *Dothudeomycetes*) and lower expression of *Basidiomycota*, mainly because of a decrease in *Agaricales* (Li X. et al., 2019). These results may imply that decreased ITS2/16s biodiversity ratios and altered bacterial-fungal inter-kingdom networks somewhat contribute to the pathogenesis of AS. The gut dysbiosis in AS may cause bowel inflammation as reflected by increased fecal calprotectin levels (Klinberg et al., 2019).

**FIGURE 3:**
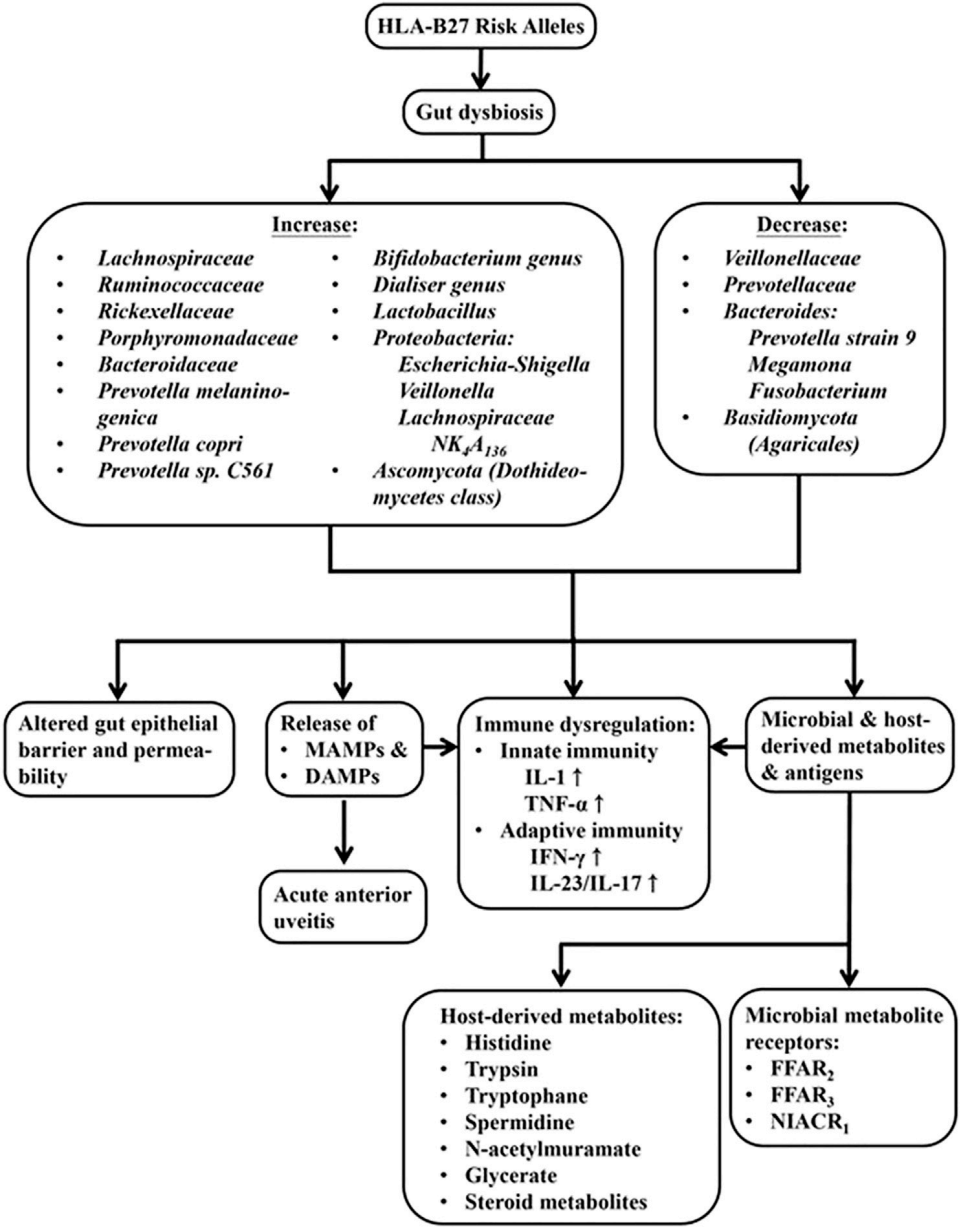
The effect of HLA-B27 risk alleles on gut dysbiosis, ensuing microbial and host metabolomic changes and immune dysregulation in the host. The dysbiosis may result in the alteration of gut epithelial barrier and its permeability, release of microbe-associated molecular pattern (MAMPs), damage-associated molecular patterns (DAMPs) from hosts, and microbial as well as host-derived metabolites. All of these molecules can elicit abnormal innate and adaptive immune responses in patients with AS.

Since HLA-B27-associated SpA is relevant to an altered gut microbiota and bowel inflammation, it is interesting to search for the cause-effect relationships among HLA-B27, gut dysbiosis and host/bacterial metabolites. Sufficient evidence indicates HLA alleles (HLA-B27 and HLA-DRB1) can affect gut microbiota as reported in rat models of SpA and RA (Asquith et al., 2017; Gill et al., 2018) as well as human AS and RA (Asquith et al., 2019; Xu and Yin, 2019). The HLA-B27-induced gut dysbiosis and zonulin upregulation may change microbiota-derived metabolites and antigens. These changes can further alter vascular barrier of gut epithelium (Ciccia et al., 2017) and deregulate intestinal immune system to activate IL-23/IL-17, IFN, TNF-α and IL-1 expression (Gill et al., 2018) in AS patients. In rat model of SpA, both microbial and host metabolites are altered. These metabolites include amino acid, carbohydrate, xenobiotics and medium-chain fatty acid. Thus, upregulation of histidine, tyrosine, supermidine, N-acetylmuramate and glycerate can be found in HLA-B27/β2m rats. The HLA-B27 presentation is also associated with altered host expression of microbial metabolite receptor genes such as *FFAR2*, *FFAR3* and *NIACR1* (Asquith et al., 2017). The studies on fecal signatures of AS patients with either gender have further revealed differences in steroid metabolites (He et al., 2019). These experiments have shown that male-specific fecal signatures include cholestan-3-ol, tocopherol, stigmastan-3,5-diene, cholest-3-ene, cholest-4-en-6-one and 1-heptatriacotanol. In contrast, the female-specific fecal signatures are ergost-5-en-3-ol acetate and D-myo-inositol. These results may indicate gender-attributed fecal signature differences between males and females, reflecting AS gender features. Metagenome-wide association study on the alterations in the gut composition has disclosed that AS subjects harbor more bacterial species associated with carbohydrate metabolism and glycan biosynthesis in their feces than normal individuals. They also express bacterial profiles with less liability to degrade xenobiotics biologically or to synthesize and transport vitamins (Huang et al., 2020). To understand thoroughly the molecular mechanisms linking intestinal microbial dysbiosis, intestinal inflammation and Th17 immunity in patients with AS, Berlingberg et al. (2021) conducted LC-MS-based metabolomic screening and shotgun metagenomic measurements in paired colon biopsies and fecal specimens. The authors also showed significant alterations in metabolites in tryptophan pathway that can increase indole-3-acetate (IAA) and indole-3-acetaldehyde (I3Ald) in AxSpA. The shotgun metagenomics confirmed abundance of numerous enzymes involved in tryptophan metabolism such as indole pyruvate decarboxylase. These enzymes can enhance significantly the generation of IAA and I3Ald to facilitate tryptophan synthesis. Tryptophan and its metabolites in this particular gut microbiome may disturb immune functions and further expedite the development of AxSpA. Moreover, gut microbiome may evolve together with the human hosts and provide them with a myriad of molecules such as microbe-associated molecular patterns (MAMPs) to augment intestinal inflammatory processes. In association with damage-associated molecular patterns (DAMPs), the inflammatory processes may be triggered further in the already ongoing pathological status such as HLA-B27-associated acute anterior uveitis (Rosenbaum and Asquith, 2018; Lu Yang et al., 2021). These data support that the invisible “essential organs” can communicate with each other and “talk” across with host immune cells in healthy individuals as well as in AS patients. The effects of HLA-B27 risk alleles on gut microbiota and metabolomic changes in patients with AS are depicted in Figure 3.

## Immune Dysfunctions in Patients With AS

Many studies have shown that various immune-related cells, *via* their secreting cytokines and molecules, may play crucial roles in AS pathogenesis (Mei et al., 2011; Liu et al., 2015; Madej et al., 2015; Sveaas et al., 2015; Wang et al., 2016; Yang et al., 2016; Wang et al., 2018). These data indicate that both innate and adaptive immune cells are involved in AS pathogenesis. The innate immune cells include dendritic cells, macrophages and natural killer cells. The adaptive immune cells include helper T, Treg, CD8^+^T, and B cells (Rezaiemanesh, et al., 2018). Much evidence has demonstrated that certain phenotypes of resting and activated macrophages expressing scavenger receptor, CD163, can link between immune alterations of the gut and synovial inflammation in AS (Baeten et al., 2002; Slobodin et al., 2012; Talpin et al., 2014; Wright et al., 2016; Rezaiemanesh et al., 2017). The abnormal polarization of macrophages induced by IL-4 was found in AS patients (Lin et al., 2015). Besides, the studies on immune dysfunctions of T cell subpopulations are prosperous. These results revealed increased frequency of Th2 (Yang et al., 2004) and Th17 (Jandus et al., 2008; Shen et al., 2009; Xueyi et al., 2013), abnormal form of HLA-B27 expression on CD4^+^ T cells (Boyle et al., 2004), defective function of CD24^+^CD38^+^ regulatory B cells (Chen M. et al., 2016), and expansion of CD4^+^CD28^high^ Treg cells (Ciccia et al., 2010). Recently, two molecules on T lymphocytes, T cell immunoglobulin and mucin-domain-containing molecule 3 (Tim-3) and programmed death-1 (PD-1) for negative regulation of immune responses, attracted investigators to focus on AS pathogenesis (Zhou et al., 2015). PD-1 expression in T cells was reported to inversely relate to the spinal radiologic changes in Taiwanese patients with AS (Chen et al., 2011). Tim-3 polymorphism could result in down-regulation of the expression of itself and be involved in AS susceptibility (Wang et al., 2014). Furthermore, other investigations revealed a decreased expression of PD-1 on CD8^+^ T cells in AS patients. The deficiency may activate immune responses in AS patients (Duan et al., 2017). More recent investigations have disclosed that Tim-3^+^CD8^+^ and PD-1^+^CD8^+^ T cells can produce more IL-10 than other subsets.

Another studies have revealed that either low percentage (Wu et al., 2011; Zhao et al., 2011; Xueyi et al., 2013) or functional impairment (Guo et al., 2016; Wang et al., 2018) in CD4^+^ Treg may be present in AS patients. On the contrary, meta-analyses have unveiled that only the proportions of CD4^+^CD25^+^FOXP3^+^ Treg, CD4^+^CD25^high^CD127^high^ cells, or CD4^+^CD25^+^CD127^low^ cells in peripheral blood of AS patients are significantly decreased (Lai et al., 2019; Li M. et al., 2020).

Several studies have been published in recent years demonstrating the pivotal role of gut-microbiota and IL-23/IL-17 axis in the AS pathogenesis (Jandus et al., 2008; Milanez et al., 2016; Mei et al., 2011; Zeng et al., 2011; Appel et al., 2011; Singh, et al., 2011; Babaie et al., 2018). Enthesis inflammation (enthesitis) was demonstrated to be IL-23-dependent (Sherlock et al., 2012; Benham et al., 2014) and likewise IL-17-dependent (Shabgah et al., 2017). Moreover, investigations from the blood samples of AS patients revealed the abundance of Th17 (Shen et al., 2009; Zhang et al., 2012), Th22 (Zhang et al., 2012) and γ/δ T cells (Kenna et al., 2012) with high levels of IL-17 in the circulation (Wendling et al., 2007; Mei et al., 2011; Lin et al., 2015). The innate lymphoid cells (ILCs) can stimulate inflammation in the gut with respect to AS. NK*p*44^+^ ILC3 cells were found expanded in the intestine, synovial fluid, bone marrow and peripheral blood of patients with AS to produce IL-17, IL-22 (Ciccia et al., 2015), and granulocyte-macrophage colony-stimulating factor (GM-CSF) but not IL-17A in the inflamed joints (Blijdorp et al., 2019). The intraepithelial lymphocytes (IELs) are another T cell subpopulation within the intestinal epithelium in close contact with bacteria that can be affected by intestinal microbiota. The total number of IELs is significantly decreased in AS due to a decrease in TCR β^+^ IELs. These IELs can secrete increased amount of IL-1β, IL-17A and IFN-γ in Crohn’s disease and significantly enhance the amount of TNF-α in AS (Regner et al., 2018). All of these results may suggest a correlation between altered microbiota and IEL function in AS.

**FIGURE 4:**
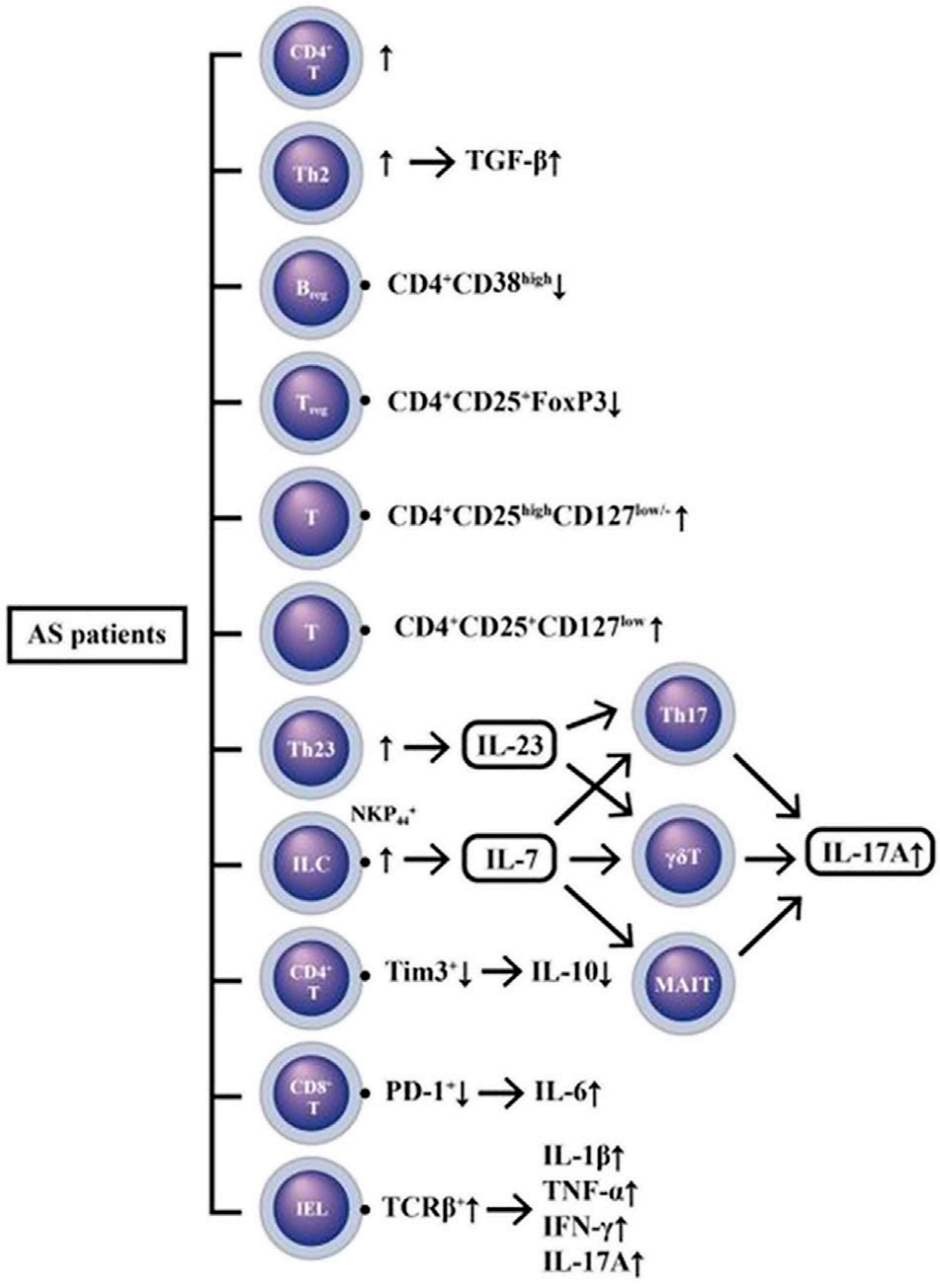
A diversity of immune cell dysfunctions with excessive innate (IL-1β, IL-6, TNF-α) and adaptive (IFN-γ and IL-17A) cytokine production in patients with AS. IL-7 released from gut innate-like lymphoid cells (ILC) can stimulate Th17 γ/δT and MAIT (mucosa-associated invariant T) to produce IL-17A. In addition, the intra-epithelial lymphocytes (IELs) are the T cells within intestinal epithelium which can also excrete IL-17A and other proinflammatory cytokines (IL-1β, TNF-α, and IFN-γ) to mediate tissue inflammation. Furthermore, both regulatory T cell (Treg) and regulatory B cell (Breg) hypofunctions may also participate in the immune dyregulation in AS patients.

The pathogenesis of AS is characterized by a predilection of adaptive immunity toward IL-23/IL-17 axis with the presence of a polarization stimulator for Th17 response. As a result, the IL-17 and TNF-α production are enhanced. However, failure of IL-23 blockade in the treatment of spinal polyenthesitis but not peripheral enthesitis has ever been encountered (McGonagle et al., 2021). Thus, the importance of IL-23 pathway in AS pathogenesis awaits further evaluations. A recent study has even demonstrated high levels of IL-7 mRNA and peptide in the peripheral type SpA (Rihl et al., 2008). IL-7 belongs to a hematopoietin cytokine family with a molecular weight of 17.4 kDa (Sutherland et al., 1989). It can stimulate Th17 cells, innate immune cells like γ/δ T cells (Michel et al., 2012) and mucosa-associated invariant T (MAIT) cells (Tang et al., 2013) to produce proinflammatory cytokines including IL-17. In addition, these innate-like T cells rather than Th17 cells have been proved to be the main source of IL-17A (Venken and Elewaut, 2015; Debusschere et al., 2016). It appears that IL-7 is more important than IL-23 in the polarization of type 17 (IL-17) signature because IL-7 receptor is present in the key cells of innate immunity that are essential for the polarization of type 3 (IL-3) response and SpA (Gonçalves and Duarte, 2019). The immune dysfunctions in patients with AS are illustrated in Figure 4.

## Pathogenesis of Enthesitis in AS Patients

Enthesis is regarded as the region where tendon attaches to bone. In broad sense, the entheseal tissues include fibrocartilage, bursa, fat pad, deeper fascia, adjacent trabecular bone networks and enthesis. These tissues play an anchorage between mobile organs and stress resistance (Benjamin and McGonagle, 2009). It has been recognized that enthesitis become the primary pathological process underlying SpA-associated skeletal inflammation (Watad et al., 2018). Normal entheseal tissues contain group 3 NK*p*44^+^ ILCs, γ/δ T cells, conventional CD4^+^ and CD8^+^ T cells, and cells of myeloid lineage. These cells are capable of producing prostaglandins, different growth factors and proinflammatory cytokines (TNF-α and IL-17) for physiological tissue repair and homeostasis (Reinhardt et al., 2016; Cuthbert et al., 2017; Cuthbert et al., 2019; Watad et al., 2020; Russel et al., 2021). In pathological condition, the IL-23-dependent γ/δ T cells can produce IL-17 that accumulates in the enthesis, aortic valve and ciliary body to cause the extra-skeletal manifestations in patient with AS (Reinhardt et al., 2016; Bridgewood et al., 2020).

**FIGURE 5:**
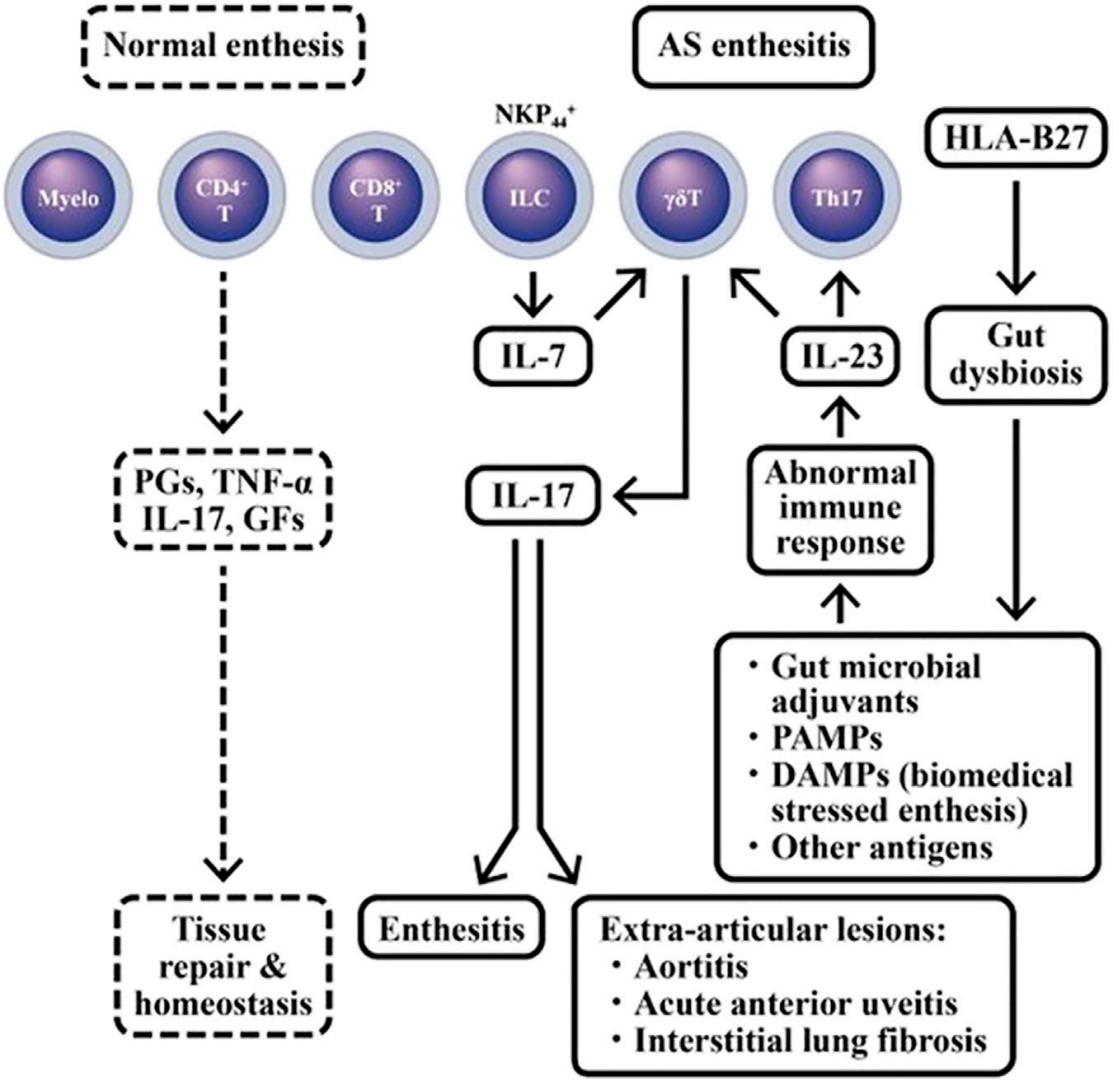
Dissection of immune-related cells in normal enthesis and AS enthesitis. In normal enthesis, many different cell populations may release physiological amount of prostaglandins (PGs), TNF-α, IL-17 and various growth factor (GFs) to maintain tissue repair and homeostasis in these biomedical stressed-entheses. However, in HLA-B27 risk allele (+) AS patients, the aberrant IL-17 production can cause inflammation in enthesis (enthesitis) as well as extra-articular manifestations such as aortitis, acute anterior uveitis, and interstitial lung fibrosis. PAMP, pathogen-associated molecular pattern; DAMP, damage-associated molecular pattern; NKP44, Natural cytotoxicity triggering receptor 2 or CD336.

Altered microbiota associated with abnormal immune responses to commensal micro-organisms may also contribute to the occurrence of enthesitis-related arthritis (Stoll et al., 2014). The “danger signals”from exogenous intestinal microbial adjuvants or pathogen-associated molecular patterns (PAMPs) can destroy “self-molecules” within the cells. Alternatively, the damage-associated molecular patterns (DAMPs) from highly biomechanically stressed entheses can disturb “fine tuning” of cytokine production in homeostatic entheseal tissues. The net-effect of these processes may serve as key drives for the onset, evolution, sustenance, flare-up, and eventual outcomes of r-AxSpA (Sharif et al., 2020). The cellular and molecular bases for enthesitis in AS patients are illustrated in Figure 5.

## Pathogenesis of Osteoporosis and Osteogenesis in AS Patients

A mystery of skeletal damage in patients with AS is the consequence of bone destruction followed by the new bone formation. The inflammation-induced osteoporosis in the spine and peripheral bones is quite common in the early stage of AS. It can cause trabecular bone weakness and lead to an increase in spinal fracture rate (Davey-Ranasinghe and Deodhar, 2013). Enhanced expression of IL-17 in the serum and synovial fluid has been reported implicating in the bone loss of AS patients (Akgöl et al., 2014). However, another puzzle of proinflammatory cytokine, IL-17 family, effecting on the bone metabolism, arises since IL-17A can enhance both bone loss and osteogenesis.

Investigations have unraveled that IL-17 can activate osteoclasts (OCs) to express RANK ligand (RANKL), which then reciprocally stimulates OCs themselves by RNAK-RANKL interaction and induces bone absorption (Page and Miossec, 2005; Miossec, 2009). IL-17A-stimulated miR214 expression in OCs is an important inhibitor for bone formation in AS patients (Wang et al., 2013; Zhao et al., 2015; Liu et al., 2020). Nevertheless, adding of exogenous IL-17A into cultured normal primary bone-derived cells (BdCs) promotes OC activity and differentiation as evidenced by increased alkaline phosphatase (ALP) activity through JAK2/STAT3 pathway (Jo et al., 2018a; Wang et al., 2018).

On the other hand, abnormal bone remodeling with excessive new bone formation can cause syndesmophytes or even “bamboo spine” to limit the spinal motility in AS patients. Many studies have revealed that higher bone morphogenetic proteins (BMP), lower Dickkopf-1 (DKK-1) levels (Liao et al., 2018), and increased ALP activity (Jo et al., 2019) in serum are parallel to the accelerated osteogenesis in the BdCs derived from AS patients (AS-BdCs) (Jo et al., 2018b; Kang et al., 2018). For investigating the relevance of ALP to the regulation of osteoblast (OB) differentiation in AS patients, ALP was inhibited in AS-BdC culture. A remarkable suppression of the master transcriptional factor in OB, RUNX2, was observed. This implies that RUNX2 can regulate promoter activity of ALP through a positive ALP-RUNX2 feedback mechanism (Jo et al., 2019). To further identify the role of HLA-B27 in syndesmophyte formation in AS, the mesenchymal stem cells (MSCs) obtained from enthesis of AS patients were studied. The results demonstrated that HLA-B27-mediated activation of the SXBP1/RARB/TNAP (tissue non-specific alkaline phosphatase) axis is essential in the development of syndesmophyte in AS patients (Liu C.-H. et al., 2019). Furthermore, the expression of miR-146a is up-regulated and *DKK1* is down-regulated respectively in capsular tissue of the hip in AS patients. Therefore, a negative correlation was displayed between the expressions of miR-146a and *DKK1*. Further investigations disclosed that miR-146a could inhibit *DKK1* expression by directly targeting 3′-UTR region of *DKK1* (Di et al., 2018). In addition, the level of miR-17-5p is significantly elevated in fibroblasts and ligament tissues, which is assumed to be targeting the 3′-UTR of ankylosis protein homolog (ANKH). This may subsequently increase osteogenesis in AS patients. Down-regulation of miR-17-5p slowed AS progression *via* regulation of DKK1 and VEGF. These findings have verified the role of miR-17-5p-ANKH axis in regulating heterotropic ossification in AS patients (Qin et al., 2019).

**FIGURE 6:**
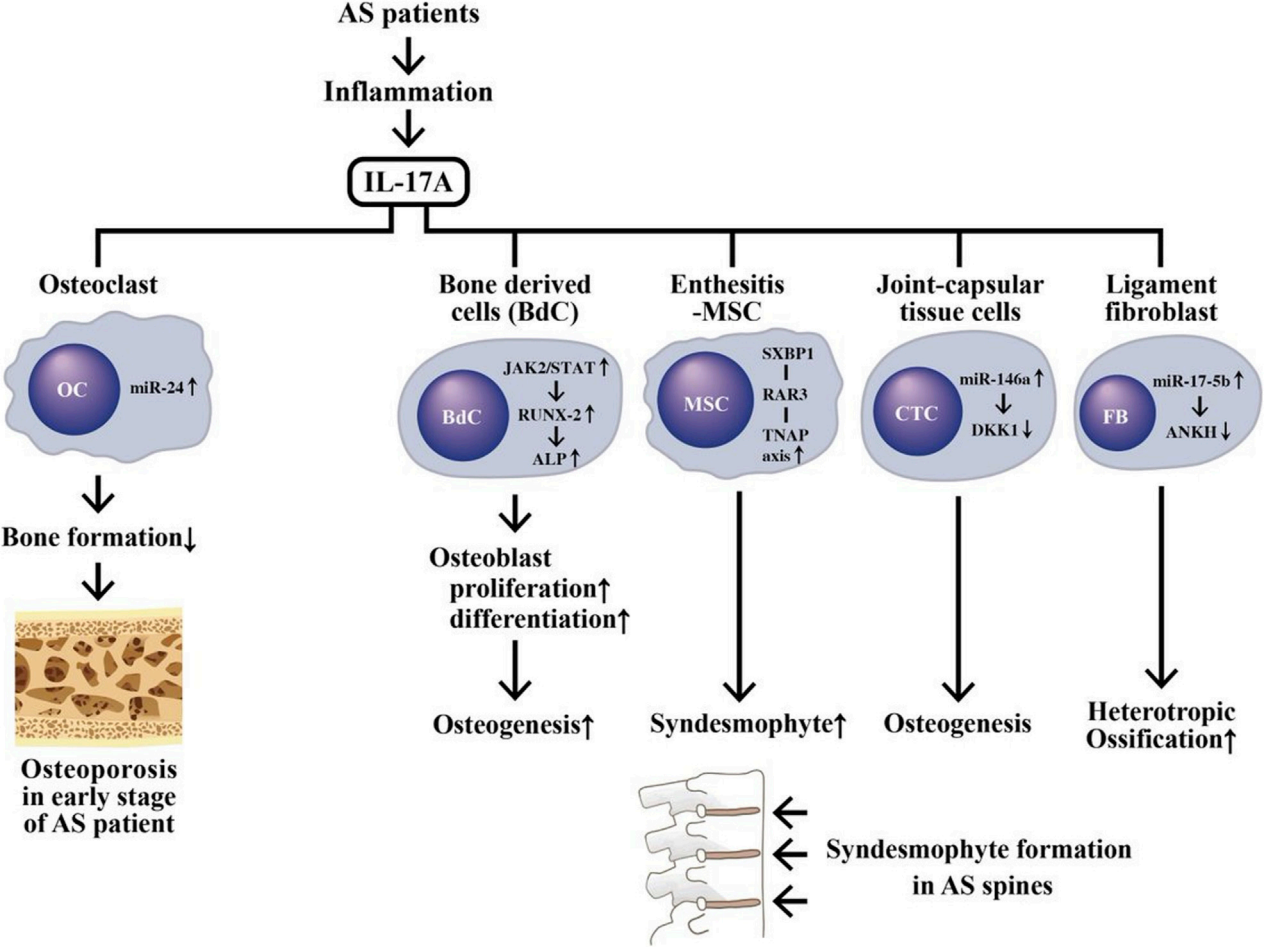
The aberrant IL-17A production during inflammation in AS patients causing a coexistence of osteoporosis in early stage of disease and new bone formation (syndesmophyte) in late stage of disease. IL-17A can activate osteoclasts (OCs) *via* stimulating miR-24 expression to suppress bone formation and subsequently result in osteoporosis. On the other hand, IL-17A can also stimulate Wnt/β-catenin, ALP/TNAP and abnormal ncRNAs expression to impede DKK1 pathways in bone-derived cells (BdC), enthesitis-derived mesenchymal stem cells (MSCs), connective tissue cells (CTCs) and ligament fibroblasts (FB). These activated cells can then induce osteogenesis and syndesmophyte formation. JAK, Janus kinase; STAT, signal transducer and activator of transcription; ANKH, Progressive ankylosis protein homolog (ANK ilosis H omolog).

The transgenic SpA-associated and non-SpA-associated HLA-B27 subtypes in *Drosophila* revealed an antagonistic interaction of HLA-B27 against activin receptor-like kinase-2 (ALK2). This antagonistic interaction may exert inhibitory effects on TGF-β1BMP signaling pathway at the cross-road between inflammation and ossification and become a putative mechanism for HLA-B27-mediated SpA development (Grandon et al., 2019). The molecular basis underlying the dilemma in inflammation-mediated bone metabolism in AS patients is shown in Figure 6.

In addition to the above mentioned factors contributing to AS pathogenesis, it is believed that the environmental factors might affect the hereditable epigenetic regulation of the down-stream gene expression in developing human diseases. These may include infectious, autoimmune/inflammatory, or neoplastic diseases. Recently, (Ghafouri-Fard et al., 2021a), have unveiled the interaction between ncRNAs and Toll-like receptors (TLRs) in transducing both MyD88-dependent and TRIF-dependent signaling cascades to induce human inflammatory and autoimmune disorders. Furthermore, the same group have found in the literature that both miRs and lncRNAs can regulate bone development processes including osteogenesis. Both intramembranous and endochondrial ossification of osteogenesis were observed (Ghafouri-Fard et al., 2021b). miRs were found to exert their actions through both Wnt/β-catenin and TGF-β/BMP pathways whereas lncRNAs worked as molecular sponge for binding miRs to directly affect these pathways and osteogenic transcription factors. The examples include MALAT1/miR-30, MALAT1/miR-214, LEF1-AS1/miR-24-3P, MCF2L-AS1/miR-33a, MSC-AS1/miR-140-5P, and KCNQ1OT1/miR-214. It is quite interesting that nuclear factor-kappa B (NF-κB) represents a group of inducible transcription factors (TFs) to regulate gene expression implicated in the immune responses. NF-κB can functionally interact with ncRNAs to construct an intricate NF-κB-miRs-lncRNAs network in regulating down-stream gene expression in different aspects. This type of network interactions among miR-146a/b, MALAT1, NKILA and NF-κB have been reported in the pathogenesis of some inflammatory conditions (Ghafouri-Fard et al., 2021c). Collectively, these data can provide some clues to support the interactions among environmental factors, gut dysbiosis, aberrant ncRNA expression and inflammation/autoimmunity in the development of AS. We are going to discuss in detail the aberrant epigenetic regulation in AS pathogenesis in the next section.

## Aberrant Epigenetic Regulation in AS

Epigenetics is a study on the heritable changes in gene expression which takes place without an alteration in DNA sequence but with modulations of chromatin-associated molecules caused by environmental factors. These environmental factors include dietary nutrition, lifestyle, exercise/physical activity, drugs/toxins and other miscellaneous contributory factors (Abdul et al., 2017; Heinbockel and Csoka, 2018; Martin and Fry, 2018). In general, epigenetic study may include DNA methylation, histone acetylation/deacetylation and circular RNA (cRNA) regulation (Berlingberg and Kuhn, 2020).

DNA methylation is an epigenetic modification with addition of methyl groups to cysteine or adenine residue to control gene transcription (Dor and Cedar, 2018). Many factors can affect DNA methylation such as age, sex, smoking, medications, alcohol and nutrition-diet (Whyte et al., 2019). DNA methyltransferase 1 (DNMT1) is an enzyme that regulates methylation of cytosine residues. Decreased DNMT1 expression can increase gene expression. Previous studies have shown that the expression level of DNMT1 in AS patients is significantly down-regulated which is associated with hypermethylation of the promoter region of *DNMT1* (Aslani et al., 2016). These results suggest that the dysregulation of DNMT1 expression *via* altered methylation level of the other target genes may contribute to AS pathogenesis. Recently, the genome-wide DNA methylation profile analysis identified many altered DNA methylation sites in the peripheral blood mononuclear cells (PBMCs) of AS patients. These sites include hypermethylation of *HLA-DQB1* (Hao et al., 2017), hypermethylation of CpG3 and CpG5 in B-cell chronic lymphocytic leukemia/lymphoma 11B (*BCL11B*) (Karami et al., 2017), and hypermethylation of GTPase-related genes (Coit et al., 2019). However, HLA-B27 bearing patients with AS were found to have some hypomethylated DNA promoters in HCP5 tubulin folding cofactor A (*TBCA*) and phospholipase D family member 6 (*PLD6*) (Coit et al., 2019).

To elucidate the epigenetic regulation of immune dysfunctions in AS, the DNA methylation profile of blood cells was analyzed. Hypermethylation of the promoter in interferon regulatory factor 8 (*IRF8*) (Chen et al., 2019) and 2CpG islands of IL-12B (*IL12B-1* and *IL12B-2*) (Zhang et al., 2019) were found. Both IFN-γ and IL-12 are crucial cytokines in suppressing Th17 cell proliferation and differentiation, which contribute in consequence to reduce severity of AS. Further investigations disclosed that hypermethylated miR-34b promoter leads to upregulation of miR-34b, thus inhibiting the IL-12B gene expression and alleviating disease activity of AS (Meng et al., 2021).

**FIGURE 7:**
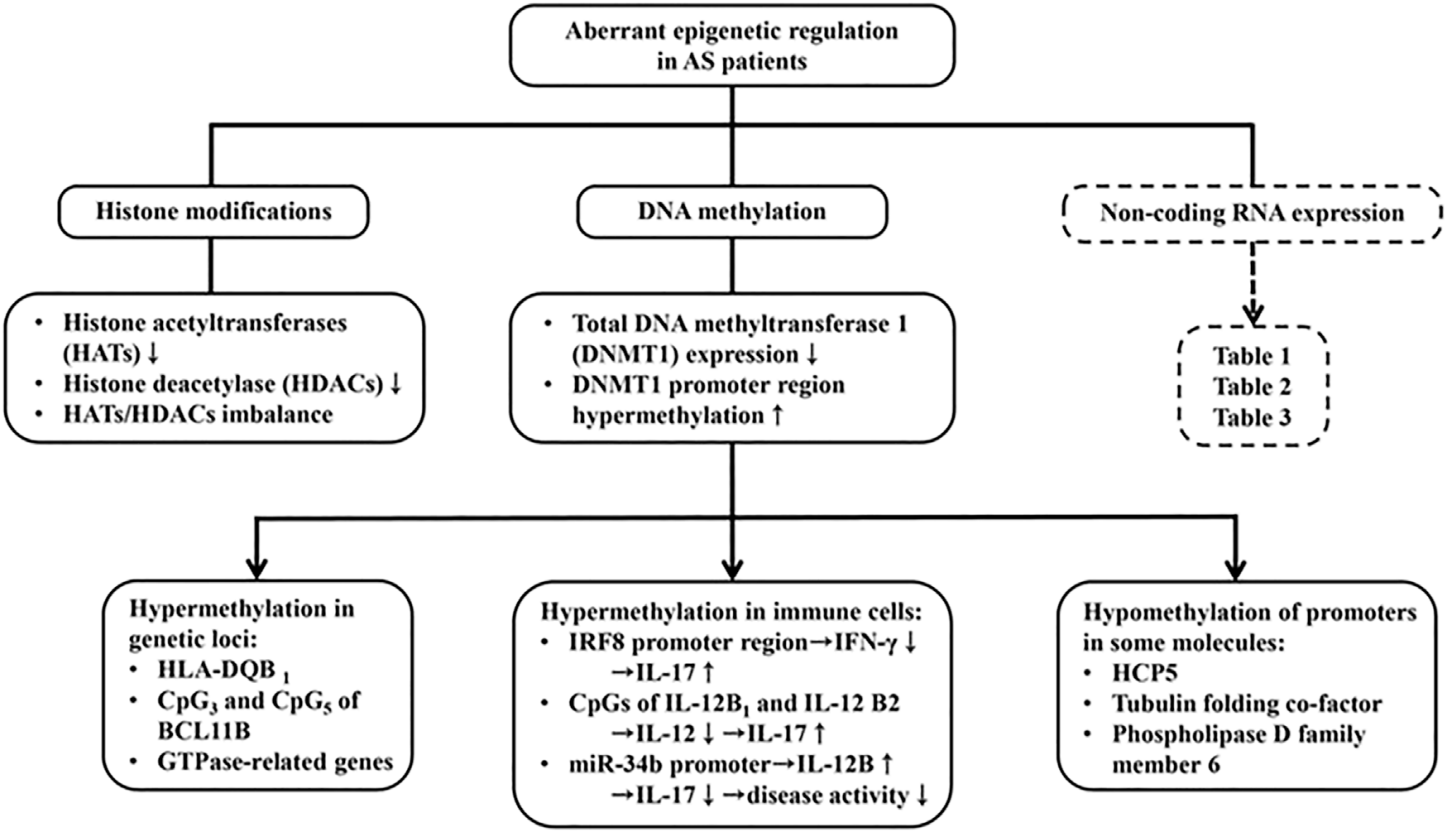
The contribution of aberrant epigenetic regulations in AS pathogenesis including histone modiciations, DNA methylation and ncRNA expression. Decreased both HATs and HDACs expression, and therefore imbalanced HATs/HDACs ratio are found in AS. Moreover, hypermethylation of HLA-DQB1, CpG3 and CpG5 isolates of BCLIIB (B cell chronic lymphocytic leukemia IIB) and GTPase-related genes are found. In contrast, DNA methyltransferase (DNMT1) promoter region hypermethylation was found in AS patients. This can cause hypermethylation in the promoter region of IFN-γ and IL-12 and conversely enhance IL-17 production. On the contrary, hypermehtylation of miR-34b promoter increase IL-12 production to suppress IL-17 expression. The abnormal ncRNA expressions in the intracellular and extracellular parts with their modes of action are presented in Tables 1–3.

On the other hand, histone modification allows activation (euchromatin) and deactivation (heterochomatin) of chromatin by two enzymes, histone acetyltransferase (HATs) and histone deacetylase (HDACs) (Allis and Jenuwein, 2016). In PBMC study, decrease in and imbalance between HAT and HDAC activities were present in AS patients, compared to the healthy controls (Toussirot et al., 2013). The aberrant DNA methylation and histone modifications in PBMC are depicted in Figure 7.

### The Characteristics of Non-Coding RNAs and Their Roles in the Pathogenesis of and Clinical Applications in AS Patients

ncRNAs are single-stranded RNAs composed of microRNAs (miRs, with 20-24 nucleotides) and long non-coding RNAs (lncRNAs, with more than 24 nucleotides and less than 300 nucleotides). They regulate gene expression and therefore are involved in physiological and pathophysiological processes. They exist in the cells, extracellular fluid and cell-derived exosomes in a stable form. In addition, lncRNAs can act as sponge for modulating miR functions. Accordingly, ncRNA expression profiles can serve as biomarkers for disease activity, pathogenesis, prognosis and therapeutic monitoring of the diseases (Mohammadi et al., 2018; Berlingberg and Kuhn, 2020; Motta et al., 2020). Besides their molecular stability, the characteristic tissue specificity, easy obtainability from different biological fluids (plasma, saliva, urine, synovial fluid and other tissue fluid), and powerful discrimination render ncRNA profiling a useful tool in studying autoimmune, inflammatory and neoplastic diseases (Park et al., 2009; Pauley et al., 2009). Therefore, we will discuss in detail the contribution of ncRNAs to the deranged T cell responses, inflammation, altered bone homeostasis and monitoring of disease activity in AS.

Lai et al. (2013) discovered that three ncRNAs, miR-16, miR-221 and let-7i, were over-expressed in T cells from AS patients (AS-T). TLR-4 has been confirmed to be the target molecule of let-7i in AS-T cells. In addition, increased expression of let-7i enhanced IFN-γ production in AS patients. miR-221 and let-7i were also associated with disease activity of lumbar spine (as calculated by BASRI) in AS. Huang et al. (2014) found that significant higher expression of miR-29 in PBMCs of AS patients, although not correlated to disease activity, could be used as a useful diagnostic biomarker in new bone formation. The same authors also found that the mRNA levels of miR-29a, *DKK-1*, β-catenin and *RUNX2* were significantly higher whereas that of *GSK-3b* was significantly lower in AS patients (Huang et al., 2017; Huang et al., 2019). These data imply that miR-29 might become a useful marker for new bone formation in AS patients as evidenced by its ability to regulate *DKK-1* in *Wnt* signaling pathway.

Fogel et al. (2019) investigated the miR expression in both CD14^+^ monocytes and CD4^+^ T lymphocytes from AS patients. The group found downregulated miR-361-3p, miR-223-3p, miR-384, and miR-16-5p in monocytes and upregulated miR-16-1-3p, miR-28-5p, miR-199a-5p, and miR-126-3p in T lymphocytes that might contribute to AS pathophysiology. Li X et al. (2019) reported elevated expression of miR-17-5p, miR-27a, miR-29a and miR-126-3p in PBMCs of axial SpA, which might be regarded as useful diagnostic markers in AS. Furthermore, Yang et al. (2019) provided evidence that miR-335-5p, miR-27a and miR-218 would predispose syndesmophyte formation in AS patients. Recently, Ni and Leng, 2020 have found that miR-495 in PBMC, whole blood, and serum is downregulated because its promoter region is highly methylated in AS patients. Besides, the miR-495 expression is negatively associated with programmed cell death protein 10, *PDCD10*. This may indicate *PDCD10* expression can be targeted by miR-495 in AS. Bioinformatic analyses and signaling pathway studies have revealed that miR-495 can down-regulate β-catenin and TGF-β1. The intracellular ncRNA expressions in immune cells (PBMCs, T cells or monocytes), their modes of action and clinical applications in AS are summarized in Table 1.

**TABLE 1:** Intracellular ncRNA expressions, their modes of action and clinical applications in the immune cells of patients with AS.

Cell/Body fluid	ncRNA	Mode of action	Biomarker	References
T cell	miR-16↑	IFN-γ↑	Disease activity in lumbar spine	Lai et al. (2013)
	miR-221↑			
	lncRNA let-7i↑			
PBMC	miR-29↑	DKK-1↑	Disease activity	Huang et al. (2014)
		β-catenin↑	Diagnostic biomarker for new bone formation	Huang et al. (2017)
		RUNX2↑		
		GSK-3β↓		
CD_14_^+^ M*ϕ*	miR-361-3p↓	AS pathogenesis		Fogel et al. (2019)
	miR-223-3p↓			
	miR-484↓			
	miR-16-5p↓			
CD_4_^+^ T	miR-16-1-3p↑			
	miR-28-5p↑			
	miR-199a-5p↑			
	miR-126-3p↑			
PBMC	miR-17-5p↑		Diagnostic biomarker	Li et al. (2019a)
	miR-27a↑			
	miR-29a↑			
	miR-126-3p↑			
PBMC	miR-335-5p		New bone formation	Yang et al. (2019)
	miR-27a			
	miR-218			
PBMC/Serum*	miR-495↓	PDCD_10_↑	New bone formation	Ni and Leng, (2020)
		β-catenin↑		
		TGF-β_1_↑		

M*ϕ*, macrophage; PBMC, peripheral blood mononuclear cell; CD, cluster of differentiation; IFN, interferon; RUNX, Runt related transcriptional factor; PDCD, programmed cell death protein; GSK, glycogen synthase kinase; TGF, transforming growth factor; DKK, Dickkopf related protein. * also appearing in serum.

The extracellular ncRNAs may include those in circulatory tissue fluid (serum, plasma, etc.) and tissue-derived (exosomes) ones. Qian et al. (2016) unraveled that serum miR-146a and miR-155 were significantly upregulated in AS. Moreover, the serum level of miR-155 is associated with disease activity and the severity of thoracolumbar kyphosis secondary to AS. Prajzlerová et al. (2017) found that miR-625-3p can reflect disease activity in AS with spinal involvement. Moreover, miR-29a-3p, miR-146a-5p and miR-222-3p are involved in extracellular matrix formation and inflammation, and are associated with spinal changes and disease activity (BASDAI) in AS patients. These dysregulated miRs are also suggestive of their potential as biomarkers for disease progression. Perez-Sanchez et al. (2018) unclosed that higher expression levels of miR-146a-5p, miR-125a-5p, miR-151a-3p and miR-22-3p and lower expression levels of miR-150-5p and miR-451a were present in the AS plasma. Bioinformatic analysis has revealed that these six miRs target proinflammatory and bone remodeling genes. Besides, miR-125a-5p, miR-151a-3p, miR-150-5p and miR-451a expression are related to the presence of syndesmophytes in AS. Accordingly, these six plasma miR signature can become gorgeous non-invasive biomarkers for AS diagnosis. Recently, Reyes-Loyola et al. (2019) assessed plasma levels of ncRNAs in Mexican AS patients. They found plasma lnc let-7 was higher in patients and might serve as a diagnostic biomarker in Mexican AS patients. On the other hand, plasma miR-16 level is inversely correlated to ASDAS-CRP score and MMP-1 level, thus, serving as disease activity marker. Li Y et al. (2020) have further found that plasma lncRNA MEG3 is downregulated and negatively correlated to the levels of IL-1β, IL-6 and TNF-α in AS patients, and can block the inflammatory response of the immune cells in AS patients. Conversely, plasma miR-146a was upregulated and positively correlated to the proinflammatory IL-1β, IL-6 and TNF-α. The authors also clarified that over expression of miR-146a could revert the inhibitory effect of abnormal MEG3 expression on inflammatory cytokines. These data imply that lncRNA MEG3 plays an anti-inflammatory role *via* targeting miR-146a and thus can provide a new potential therapeutic role for AS treatment. In further elucidation of the molecular mechanism for the therapeutic potential of lncRNA MEG3, Ma et al. (2020) found that the expression levels of *MEG3* and sclerostin (*SOST*) are decreased but lncRNA let-7 is increased in AS patients. Their results confirmed that *MEG3* can interact with (sponge) let-7i in AS fibroblast and promotes *SOST* expression to restrain the progression of AS. This would provide a new treatment modality in AS. Fotoh et al. (2020) discovered a higher expression of miR-125a and a lower expression of miR-451a in the plasma from active Egyptian AS patients. Interestingly, both miRs were able to distinguish AS patients with a structural damage and could be used as sensitive diagnostic, prognostic and disease burden biomarkers for AS patients. The extracellular (serum or plasma) ncRNA expression, their modes of action and clinical application in AS patients are listed in Table 2.

**TABLE 2:** Extracellular (serum, plasma) ncRNA expressions, their modes of action and clinical applications in patients with AS.

Source	ncRNA	Mode of action	Biomarker	References
Serum	miR-146a↑		Disease activity and kyphosis	Qian et al. (2016)
	miR-155↑			
	miR-625-3p		Disease activity and	Prajzlerová et al. (2017)
			Spine involvement	
Serum	miR-29a-3p	Extracellular matrix formation and inflammation	Disease activity and disease progression	
	miR-146a-5p			
	miR-222-3p			
Plasma	miR-146a-3p↑	Target inflammatory and bone remodeling	Diagnostic and new bone formation	Perez-Sanchez et al. (2018)
	miR-125a-5p↑			
	miR-151a-3p↑			
	miR-22-3p↑			
	miR-150-5p↓			
	miR-451a↓			
Plasma (Mexico)	lncRNA let-7↑	MMP-1↑, CRP↑	Diagnostic	Reyes-Loyola et al. (2019)
	miR-16↓		Disease activity	
Plasma	lncRNA MEG_3_↓	IL-1β↑, IL-6↑	Disease activity	Li et al. (2020b)
	miR-146a↑	TNF-α↑		
Plasma (Egypt)	miR-125a↑	Structural damage	Diagnostic, prognostic and disease burden	Fotoh et al. (2020)
	miR-451a↓			

IL, interleukin; MMP, matrix metalloprotein; CRP, C-reactive protein; TNF, tumor necrosis factor.

In summary, these aberrant soluble extracellular ncRNAs expression in AS patients can be divided into three categories in clinical practice as shown below;

1) Biomarkers for disease diagnosis: lncRNA let-7, miR-146a-3P, miR-125a-5P, miR-151a-3P, miR-22-3P, miR-150-5P, and miR-451a.
2) Biomarkers for disease activity: miR-146a, miR-155, miR-625-3P, miR-29a-3P, miR-146a-5P, miR-222-3P, and lncRNA-MEG.
3) Biomarkers for both diagnosis and disease activity: miR-16a, miR-146a, miR-125a, and miR-451a

### The Role of ncRNAs in Enthesitis and Ligament Inflammation

Enthesis is the characteristic sites where pathological processes occur in AS patients, causing enthesitis. To reflect ossifications more realistically in the ligaments of AS patients, the epigenetic regulation of the cultured ligament-derived fibroblasts were analyzed for osteogenic differentiation. Zhang et al. (2017) compared miR, lncRNA and mRNA profiles in hip joint ligament tissues from AS patients. The authors identified that miR-17-5p and miR-27b-3p could increase the potentials of osteogenic differentiation in ligament fibroblasts of the hip joint. Tang et al. (2018) isolated ligament fibroblasts from AS patients and induced them to differentiate into osteoblast (OB). During osteogenic differentiation, miR-124, β-catenin, osteorix and *RUNX2* expression gradually increased, while that of GSK-3β gradually declined. Zhao et al. (2020) have demonstrated that miR-204-5p can negatively regulate *NOTCH* 2 expression in osteogenic differentiation in the ligament fibroblasts derived from AS patients *in vitro*. These results may provide a therapeutic basis for the effective treatment for patients with AS. In the OBs isolated from murine model of AS, Ma et al. (2019) found that miR-96 expressed at a high level while sclerostin (*SOST*) expressed at a low level. Actually, miR-96 was observed to target and negatively regulate *SOST*. Furthermore, the over-expressed miR-96 activated the *Wnt* signaling pathway and increased proinflammatory cytokines (IL-6, TNF-α, and IL-10), ALP activity, calcium nodule formation and OB viability. These results indicated that the overexpression of miR-96 can enhance OB differentiation and subsequent bone formation in AS mice *via Wnt* signaling pathway. In contrast (Liu W. et al., 2019), showed that mesenchymal stem cells (MSCs) derived from AS patients exhibited a strong capacity to inhibit osteoclastogenesis and secreted more CXCL5. Further studies showed that down-regulation of miR-4284 in AS-MSCs resulted in increased CXCL5, indicating that osteoclastogenesis may be markedly suppressed *via* miR- 4284/CXCL5 axis. By experiments with human fibroblast-like synovial cells (HFLSs) isolated from AS tissues, Du et al. (2019) showed that miR-495 and dishevelled 2 (DVL-2) molecule were negatively correlated with each other in AS. Both molecules can inhibit inflammation by down-regulating proinflammatory cytokines, IL-1, IL-6 and TNF-α and facilitate bone differentiation by up-regulating osteoprotegerin (OPG) and RANKL levels in HFLS. Besides, miR-495 and siRNA, si-DVL-2, enhanced expression of *wnt3a*, *RUNX2* and β-catenin and reduced β-catenin phosphorylation. Collectively, miR-495 depresses inflammatory responses and promotes bone differentiation of HFLSs *via* Wnt/β-catenin/RUNX-2 pathway by targeting DVL-2.

In addition to miRs and lncRNAs, circular RNAs (circRNAs) are a particular class of endogenous ncRNAs with a covalently closed circular structure (Hsu and Coca-Prados, 1979; Hansen et al., 2013; Memczak et al., 2013). Different from the linear RNAs, circRNAs lack free 3′-end poly A tail and 5′-end cap which prevent them from being degraded by nucleic acid endonuclease (Qu et al., 2015). Accordingly, the closed circular structure of circRNAs makes them extremely stable in the extracellular milieu and able to regulate the expression of target miRs by their sponge effects in human diseases (SanterBar and Thum, 2019; Wang et al., 2019). Kou et al. (2020) analyzed the circRNA expression profile of the spinal ligament tissue in AS patients. The authors found 57 circRNAs were up-regulated and 66 were down-regulated which were mainly involved in the regulation of biological processes of peptidyl-serine phosphorylation and immune system relevant to AS pathogenesis. In addition, the circRNA-miR interactions may provide new clues for understanding the mechanisms, diagnosis and potential molecular targets for the treatment in AS patients.

Another interesting findings by Tabrizi et al. (2017) were from the investigations on expression levels of the maturing microprocessor complex of miR in PBMCs from AS patients. It is believed that major enzymes responsible for miR maturation are Dicer, Drosha, and Drosha assistant DGCR8. Their results revealed that both Dicer and DGCR8 mRNA expression were down-regulated whereas Drosha mRNA expression was not influenced in AS. These data suggest that the down-regulated miR maturation components may probably contribute to the pathogenesis of AS. The intracellular expression of ncRNAs in ligament-derived fibroblasts of AS patients and their modes of action are summarized in Table 3.

**TABLE 3:** Intracellular expressions of ncRNAs in ligament-derived fibroblast, osteoblasts, bone marrow-derived mesenchymal stem cells (MSCs) and human fibroblast-like synovial cells (HFLSs) from AS patients.

Source	ncRNA	Mode of action	References
Fibroblast	miR-17-5p↑	Osteogenic differentiation↑	Zhang et al. (2017)
	miR-27b-3p↑		
Fibroblast	miR-124↑	β-catenin↑	Tang et al. (2018)
		Osterix↑	
		RUNX2↑	
		GSK-3β↓	
Fibroblast	miR-204-5p↑	Notch 2 expression↓	Zhao et al. (2020)
		Ostoegenic differentiation↑	
Murine AS osteoblast	miR-96↑	IL-6↑	Ma et al. (2019)
		TNF-α↑	
		IL-10↑	
		*Wnt* signaling↑	
		ALP↑	
		Calcium↑	
		Osteoblast viability↑	
		*SOST*↑	
		New bone formation↑	
MSC	miR-4284↓	Osteoclastogenesis↓	Liu W et al. (2019)
		Osteogenesis↑	
		CXCL5↑	
HFLS	miR-495↑	Inflammation↓	Du et al. (2019)
	DVL-2↓	IL-1↓	
		IL-6↓	
		TNF-α↓	
		Osteoprotegerin↑	
		Wnt/β-catenin/*RUNX2*↑	
		RANKL↑	

RUNX, Runt related transcriptional factor; TNF, tumor necrosis factor; IL, interleukin; ALP, alkaline phosphotase; *Wnt*, wingless and Int-1; *SOST*, sclerostin gene; CXCL, ligand for cysteine-X-cysteine chemokine; RANKL, Receptor activator of nuclear factor kappa-Β ligand; GSK, glycogen synthase kinase. DVL, segment polarity protein dishevelled homolog.

## Conclusion and Perspectives

Genetic and environmental factors intriguingly interact with each other in affecting epigenetic modifications in patients with AS. More than 100 genes have been identified to contribute to AS susceptibility. Among them, HLA-B27 subtypes, polymorphic ERAP, and IL-23R mutation seem to be significantly associated. It is also conceivable that the microtrauma in the entheses may trigger the onset of AS. Besides, the arthritogenic peptides (misfolded HLA-B27 antigen and HLA-B27 homodimer), gut dysbiosis, abnormal intestinal metabolomic products, and immune plasticity can also induce soft tissue, articular and extra-articular inflammation. The inflammation-induced osteoporosis, the subsequent osteogenesis, and finally the new bone formation may cause skeletal disability. Extra-musculoskeletal manifestations of AS include mainly anterior uveitis, aortitis and interstitial fibrosis of the upper lungs. The florid immune dysfunctions of innate and adaptive immune responses resulted from the abnormal IL-23/IL-17 axis is paramount crucial. Nevertheless, the aberrant presentations of up-stream epigenetic regulatory mechanisms are the culprits of similar importance for the abnormal immune regulation in AS. That is to say, these abnormalities of ncRNAs (i.e., miRs, lncRNAs and circRNAs) and microRNA maturing microprocessor complex (Dicer and Drosha) in PBMCs, serum/plasma and tissues play a crucial role in disrupting innate and adaptive immune responses implicated in multiple pathological processes in patients with AS. Although, the molecular mechanisms for these characteristic pathogenic events including enthesitis, osteoporosis, osteogenesis, anterior uveitis, aotitis and interstitial lung fibrosis have been discussed in detail above, the real causes of AS pathogenesis remain elusive. Some perspective investigations helpful for the understanding of this complicated epigenetic regulation are suggested:

1) A molecular basis for the induction of gut dysbiosis by HLA-B27 subtypes with its subsequent abnormal epigenetic regulation and immune dysregulation should be clarified.
2) Clinically applicable serum ncRNAs as biomarkers for measuring disease activity and assessing therapeutic response in AS patients should be identified on the level of high sensitivity and specificity, compared to the nonspecific serum CRP and stool calprotectin currently available.
3) The role of aberrant ncRNA expression with subsequent abnormal immune responses in stressful enthesis induced by denatured hyaluronan-I needs to be explored.
4) Molecular and cellular bases for the absence of rheumatoid factors in AS patients that may be relevant to deranged ncRNA expression need to be unveiled
5) The production of anti-CD74 autoantibody with its immunopathogical roles in AS patients that may be relevant to aberrant ncRNA expression should also be delineated.

## References

[B1] Abdul Q. A. Yu B. P. Chung H. Y. Jung H. A. Choi J. S. (2017). Epigenetic Modifications of Gene Expression by Lifestyle and Environment. Arch. Pharm. Res. 40, 1219–1237. 10.1007/s12272-017-0973-3 29043603

[B2] Akgöl G. Kamanli A. Ozgocmen S. (2014). Evidence for Inflammation-Induced Bone Loss in Non-radiographic Axial Spondyloarthritis. Rheumatology 53, 497–501. 10.1093/rheumatology/ket385 24262756

[B3] Akkoc N. Khan M. A. (2005). Overestimation of the prevalence of ankylosing spondylitis in the Berlin study: Comment on the article by Braun et al. Arthritis Rheum. 52, 4048–4049. 10.1002/art.21492 16320356

[B4] Aldhamen Y. A. Seregin S. S. Rastall D. P. Aylsworth C. F. Pepelyayeva Y. Busuito C. J. (2013). Endoplasmic Reticulum Aminopeptidase-1 Functions Regulate Key Aspects of the Innate Immune Response. PLoS One 8, e69539. 10.1371/journal.pone.0069539 23894499 PMC3722114

[B5] Aldhamen Y. A. Pepelyayeva Y. RastallSeregin D. P. W. S. S. Seregin S. S. Zervoudi E. Koumantou D. (2015). Autoimmune Disease-Associated Variants of Extracellular Endoplasmic Reticulum Aminopeptidase 1 Induce Altered Innate Immune Responses by Human Immune Cells. J. Innate Immun. 7, 275–289. 10.1159/000368899 25591727 PMC4417058

[B6] Allis C. D. Jenuwein T. (2016). The Molecular Hallmarks of Epigenetic Control. Nat. Rev. Genet. 17, 487–500. 10.1038/nrg.2016.59 27346641

[B7] Appel H. Maier R. Wu P. Scheer R. Hempfing A. Kayser R. (2011). Analysis of IL-17+ Cells in Facet Joints of Patients with Spondyloarthritis Suggests that the Innate Immune Pathway Might Be of Greater Relevance Than the Th17-Mediated Adaptive Immune Response. Arthritis Res. Ther. 13, R95. 10.1186/ar3370 21689402 PMC3218910

[B8] Aslani S. Mahmoudi M. Garshasbi M. Jamshidi A. R. Karami J. Nicknam M. H. (2016). Evaluation of DNMT1 Gene Expression Profile and Methylation of its Promoter Region in Patients with Ankylosing Spondylitis. Clin. Rheumatol. 35, 2723–2731. 10.1007/s10067-016-3403-x 27637577

[B9] Asquith M. Davin S. Stauffer P. Michell C. Janowitz C. Lin P. (2017). Intestinal Metabolites Are Profoundly Altered in the Context of HLA-B27 Expression and Functionally Modulate Disease in a Rat Model of Spondyloarthritis. Arthritis Rheumatol. 69, 1984–1995. 10.1002/art.40183 28622455 PMC5623151

[B10] Asquith M. Sternes P. R. Costello M. E. Karstens L. Diamond S. Martin T. M. (2019). HLA Alleles Associated with Risk of Ankylosing Spondylitis and Rheumatoid Arthritis Influence the Gut Microbiome. Arthritis Rheumatol. 71, 1642–1650. 10.1002/art.40917 31038287

[B11] Babaie F. Hasankhani M. Mohammadi H. Safarzadeh E. Rezaiemanesh A. Salimi R. (2018). The Role of Gut Microbiota and IL-23/IL-17 Pathway in Ankylosing Spondylitis Immunopathogenesis: New Insights and Updates. Immunol. Lett. 196, 52–62. 10.1016/j.imlet.2018.01.014 29409751

[B12] Babaie F. Mohammadi H. Hemmatzadeh M. Ebrazeh M. Torkamandi S. Yousefi M. (2020). Evaluation of ERAP1 Gene Single Nucleotide Polymorphisms in Immunomodulation of Pro-inflammatory and Anti-inflammatory Cytokines Profile in Ankylosing Spondylitis. Immunol. Lett. 217, 31–38. 10.1016/j.imlet.2019.10.016 31711818

[B13] Baeten D. Demetter P. Cuvelier C. A. Kruithof E. van Damme N. de Vos M. (2002). Macrophages Expressing the Scavenger Receptor CD163: a Link between Immune Alterations of the Gut and Synovial Inflammation in Spondyloarthropathy. J. Pathol. 196, 343–350. 10.1002/path.1044 11857499

[B14] Benham H. Rehaume L. M. Hasnain S. Z. Velasco J. Baillet A. C. Ruutu M. (2014). Interleukin-23 Mediates the Intestinal Response to Microbial β-1,3-Glucan and the Development of Spondyloarthritis Pathology in SKG Mice. Arthritis Rheumatol. 66, 1755–1767. 10.1002/art.38638 24664521

[B15] Benjamin M. McGonagle D. (2009). The Enthesis Organ Concept and its Relevance to the Spondyloarthropathies. Adv. Exp. Med. Biol. 649, 57–70. 10.1007/978-1-4419-0298-6_4 19731620

[B16] Berlingberg A. Kuhn K. A. (2020). Molecular Biology Approaches to Understanding Spondyloarthritis. Rheum. Dis. Clin. North. Am. 46, 203–211. 10.1016/j.rdc.2020.01.001 32340696 PMC7709878

[B17] Berlingberg A. J. Regner E. H. Stahly A. Brar A. Reisz J. A. Gerich M. E. (2021). Multi’Omics Anlaysis of Intestinal Tissue in Ankylosing Spondylitis Identifies Alterations in the Tryptophan Metabolism Pathway. Front. Immunol. 12, 587119. 10.3389/fimmu.2021.587119 33746944 PMC7966505

[B18] Blijdorp I. C. J. Menegatti S. Mens L. J. J. Sande M. G. H. Chen S. Hreggvidsdottir H. S. (2019). Expansion of Interleukin‐22- and Granulocyte-Macrophage Colony‐Stimulating Factor-Expressing, but Not Interleukin‐17A-Expressing, Group 3 Innate Lymphoid Cells in the Inflamed Joints of Patients with Spondyloarthritis. Arthritis Rheumatol. 71, 392–402. 10.1002/art.40736 30260078 PMC6519165

[B19] Boyle L. H. Goodall J. C. Opat S. S. Gaston J. S. H. (2001). The Recognition of HLA-B27 by Human CD4+ T Lymphocytes. J. Immunol. 167, 2619–2624. 10.4049/jimmunol.167.5.2619 11509603

[B20] Boyle L. Goodall J. Gaston J. (2004). The Recognition of Abnormal Forms of HLA-B27 by CD4+ T Cells. Cmm 4, 51–58. 10.2174/1566524043479257 15011959

[B21] Bridgewood C. Sharif K. Sherlock J. Watad A. McGonagle D. (2020). Interleukin‐23 Pathway at the Enthesis: The Emerging story of Enthesitis in Spondyloarthropathy. Immunol. Rev. 294, 27–47. 10.1111/imr.12840 31957051

[B22] Cardoneanu A. Mikai C. Rezus E. Burlui A. Popa I. Prelipcean C. C. (2021). Gut Microbiota Changes in Inflammatory Bowel Diseases and Ankylosing Spondylitis. J. Gastrointestin Liver Dis. 30, 46–54. 10.15403/jgld-2823 33548121

[B23] Chen M.-H. Chen W.-S. Lee H.-T. Tsai C.-Y. Chou C.-T. (2011). Inverse Correlation of Programmed Death 1 (PD-1) Expression in T Cells to the Spinal Radiologic Changes in Taiwanese Patients with Ankylosing Spondylitis. Clin. Rheumatol. 30, 1181–1187. 10.1007/s10067-011-1721-6 21547439

[B24] Chen L. Ridley A. Hammitzsch A. Al-Mossawi M. H. Bunting H. Georgiadis D. (2016a). Silencing or Inhibition of Endoplasmic Reticulum Aminopeptidase 1 (ERAP1) Suppresses Free Heavy Chain Expression and Th17 Responses in Ankylosing Spondylitis. Ann. Rheum. Dis. 75, 916–923. 10.1136/annrheumdis-2014-206996 26130142 PMC4853590

[B25] Chen M. Zhang L. Ren Y. Zhang K. Yang Y. Fang Y. (2016b). Defective Function of CD24+CD38+ Regulatory B Cells in Ankylosing Spondylitis. DNA Cel Biol. 35, 88–95. 10.1089/dna.2015.3046 26556289

[B26] Chen M. Wu M. Hu X. Yang J. Han R. Ma Y. (2019). Ankylosing Spondylitis Is Associated with Aberrant DNA Methylation of IFN Regulatory Factor 8 Gene Promoter Region. Clin. Rheumatol. 38, 2161–2169. 10.1007/s10067-019-04505-5 30900036

[B27] Ciccia F. Accardo-Palumbo A. Giardina A. Di Maggio P. PrincipatoBombardieri A. M. Bombardieri M. (2010). Expansion of Intestinal CD4+CD25highTreg Cells in Patients with Ankylosing Spondylitis: A Putative Role for Interleukin-10 in Preventing Intestinal Th17 Response. Arthritis Rheum. 62, 3625–3634. 10.1002/art.27699 20722024

[B28] Ciccia F. Guggino G. Rizzo A. Saieva L. Peralta S. Giardina A. (2015). Type 3 Innate Lymphoid Cells Producing IL-17 and Il-22 Are Expanded in the Gut, in the Peripheral Blood, Synovial Fluid and Bone Marrow of Patients with Ankylosing Spondylitis. Ann. Rheum. Dis. 74, 1739–1747. 10.1136/annrheumdis-2014-206323 25902790

[B29] Ciccia F. Guggino G. Rizzo A. Alessandro R. Luchetti M. M. Milling S. (2017). Dysbiosis and Zonulin Upregulation Alter Gut Epithelial and Vascular Barriers in Patients with Ankylosing Spondylitis. Ann. Rheum. Dis. 76, 1123–1132. 10.1136/annrheumdis-2016-210000 28069576 PMC6599509

[B30] Coit P. Kaushik P. Caplan L. Kerr G. S. Walsh J. A. Dubreuil M. (2019). Genome-wide DNA Methylation Analysis in Ankylosing Spondylitis Identifies HLA-B*27 Dependent and Independent DNA Methylation Changes in Whole Blood. J. Autoimmun. 102, 126–132. 10.1016/j.jaut.2019.04.022 31128893

[B31] Cortes A. Pulit S. L. Leo P. J. Pointon J. J. Robinson P. C. Weisman M. H. (2015). Major Histocompatibility Complex Associations of Ankylosing Spondylitis Are Complex and Involve Further Epistasis with ERAP1. Nat. Commun. 6, 7146. 10.1038/ncomms8146 25994336 PMC4443427

[B32] Costello M.-E. Ciccia F. Willner D. Warrington N. Robinson P. C. Gardiner B. (2015). Brief Report: Intestinal Dysbiosis in Ankylosing Spondylitis. Arthritis Rheumatol. 67, 686–691. 10.1002/art.38967 25417597

[B33] Cuthbert R. J. Fragkakis E. M. Dunsmuir R. Li Z. Coles M. Marzo-Ortega H. (2017). Brief Report: Group 3 Innate Lymphoid Cells in Human Enthesis. Arthritis Rheumatol. 69, 1816–1822. 10.1002/art.40150 28511289

[B34] Cuthbert R. J. Watad A. Fragkakis E. M. Dunsmuir R. Loughenbury P. Khan A. (2019). Evidence that Tissue Resident Human Enthesis γδT-cells Can Produce IL-17A Independently of IL-23R Transcript Expression. Ann. Rheum. Dis. 78, 1559–1565. 10.1136/annrheumdis-2019-215210 31530557 PMC6837256

[B35] Davey-Ranasinghe N. Deodhar A. (2013). Osteoporosis and Vertebral Fractures in Ankylosing Spondylitis. Curr. Opin. Rheumatol. 25, 509–516. 10.1097/bor.0b013e3283620777 23719363

[B37] Debusschere K. Lories R. J. Elewaut D. (2016). MAIT Cells: Not Just Another brick in the wall. Ann. Rheum. Dis. 75, 2057–2059. 10.1136/annrheumdis-2016-209695 27474762

[B38] Di G. Kong L. Zhao Q. Ding T. (2018). MicroRNA-146a Knockdown Suppresses the Progression of Ankylosing Spondylitis by Targeting Dickkopf 1. Biomed. Pharmacother. 97, 1243–1249. 10.1016/j.biopha.2017.11.067 29145150

[B39] Dor Y. Cedar H. (2018). Principles of DNA Methylation and Their Implications for Biology and Medicine. The Lancet 392, 777–786. 10.1016/s0140-6736(18)31268-6 30100054

[B40] Du W. Yin L. Tong P. Chen J. Zhong Y. Huang J. (2019). MiR-495 Targeting Dvl-2 Represses the Inflammatory Response of Ankylosing Spondylitis. Am. J. Transl. Res. 11, 2742–2753. 31217850 PMC6556642

[B41] Duan Z. Gui Y. Li C. Lin J. Gober H.-J. Qin J. Li D. Wang L. (2017). The immune dysfunction in ankylosing spondylitis patients. Bst 11, 69-76. 10.5582/bst.2016.01171 28179599

[B42] El Maghraoui A. (2011). Extra-articular Manifestations of Ankylosing Spondylitis: Prevalence, Characteristics and Therapeutic Implications. Eur. J. Intern. Med. 22, 554–560. 10.1016/j.ejim.2011.06.006 22075279

[B43] Evans D. M. Spencer C. C. Pointon J. J. Su Z. Harvey D. Kochan G. (2011). Interaction between ERAP1 and HLA-B27 in Ankylosing Spondylitis Implicates Peptide Handling in the Mechanism for HLA-B27 in Disease Susceptibility. Nat. Genet. 43, 761–767. 10.1038/ng.873 21743469 PMC3640413

[B44] Faham M. Carlton V. Moorhead M. Zheng J. Klinger M. Pepin F. (2017). Discovery of T Cell Receptor β Motifs Specific to HLA-B27-Positive Ankylosing Spondylitis by Deep Repertoire Sequence Analysis. Arthritis Rheumatol. 69, 774–784. 10.1002/art.40028 28002888

[B45] Fitzgerald G. Gallagher P. O’Shea F. D. (2020). Multimorbidity in Axial Spondyloarthropathy and its Association with Disease Outcomes: Results from the Ankylosing Spondylitis Registry of Ireland Cohort. J. Rheumatol. 47, 218–226. 10.3899/jrheum.181415 31092715

[B46] Fogel O. Bugge Tinggaard A. Fagny M. Sigrist N. Roche E. Leclere L. (2019). Deregulation of microRNA Expression in Monocytes and CD4+ T Lymphocytes from Patients with Axial Spondyloarthritis. Arthritis Res. Ther. 21, 51. 10.1186/s13075-019-1829-7 30755244 PMC6373047

[B47] Fotoh D. S. Noreldin R. I. Rizk M. S. Elsabaawy M. M. Esaily H. A. (2020). miR-451a and miR-125a Expression Levels in Ankylosing Spondylitis: Impact on Disease Diagnosis, Prognosis, and Outcomes. J. Immunol. Res. 2020, 2180913. 10.1155/2020/2180913 33426087 PMC7781682

[B48] Gao S. Xu T. Liang W. Xun C. Deng Q. Guo H. (2020). Association of Rs27044 and Rs30187 Polymorphisms in Endoplasmic Reticulum Aminopeptidase 1 Gene and Ankylosing Spondylitis Susceptibility: A Meta‐analysis. Int. J. Rheum. Dis. 23, 499–510. 10.1111/1756-185x.13795 31984677

[B49] Ghafouri-Fard S. Abak A. Shoorei H. Talebi S. F. Mohaqiq M. Sarabi P. (2021a). Interaction between Non-coding RNAs and Toll-like Receptors. Biomed. Pharmacother. 140, 111784. 10.1016/j.biopha.2021.111784 34087695

[B50] Ghafouri-Fard S. Abak A. Tavakkoli Avval S. Rahmani S. Shoorei H. Taheri M. (2021b). Contribution of miRNAs and lncRNAs in Osteogenesis and Related Disorders. Biomed. Pharmacother. 142, 111942. 10.1016/j.biopha.2021.111942 34311172

[B51] Ghafouri-Fard S. Abak A. Fattahi F. Hussen B. M. Bahroudi Z. Shoorei H. (2021c). The Interaction between miRNAs/lncRNAs and Nuclear Factor-Κb (NF-Κb) in Human Disorders. Biomed. Pharmacother. 138, 111519. 10.1016/j.biopha.2021.111519 33756159

[B52] Gill T. Asquith M. Brooks S. R. Rosenbaum J. T. Colbert R. A. (2018). Effects of HLA-B27 on Gut Microbiota in Experimental Spondyloarthritis Implicate an Ecological Model of Dysbiosis. Arthritis Rheumatol. 70, 555–565. 10.1002/art.40405 29287307 PMC6101666

[B53] Gonçalves R. S. G. Duarte A. L. B. P. (2019). IL-7 Is a Key Driver Cytokine in Spondyloarthritis? J. Immunol. Res. 2019, 1–7. 10.1155/2019/7453236 PMC6560328 31276000

[B54] Goto Y. Ogawa K. Hattori A. Tsujimoto M. (2011). Secretion of Endoplasmic Reticulum Aminopeptidase 1 Is Involved in the Activation of Macrophages Induced by Lipopolysaccharide and Interferon-γ. J. Biol. Chem. 286, 21906–21914. 10.1074/jbc.m111.239111 21531727 PMC3122245

[B55] Gracey E. Yao Y. Qaiyum Z. Lim M. Tang M. Inman R. D. (2020). Altered Cytotoxicity Profile of CD 8+ T Cells in Ankylosing Spondylitis. Arthritis Rheumatol. 72, 428–434. 10.1002/art.41129 31599089

[B56] Grandon B. Rincheval-Arnold A. Jah N. Corsi J.-M. Araujo L. M. Glatigny S. (2019). HLA-B27 Alters BMP/TGFβ Signalling in Drosophila, Revealing Putative Pathogenic Mechanism for Spondyloarthritis. Ann. Rheum. Dis. 78, 1653–1662. 10.1136/annrheumdis-2019-215832 31563893

[B57] Guo H. Zheng M. Zhang K. Yang F. Zhang X. Han Q. (2016). Functional Defects in CD4+ CD25high FoxP3+ Regulatory Cells in Ankylosing Spondylitis. Sci. Rep. 6, 37559. 10.1038/srep37559 27901054 PMC5128857

[B58] Hansen T. B. Jensen T. I. Clausen B. H. Bramsen J. B. Finsen B. Damgaard C. K. (2013). Natural RNA Circles Function as Efficient microRNA Sponges. Nature 495, 384–388. 10.1038/nature11993 23446346

[B59] Hanson A. L. Cuddihy T. Haynes K. Loo D. Morton C. J. Oppermann U. (2018). Genetic Variants in ERAP1 and ERAP2 Associated with Immune-Mediated Diseases Influence Protein Expression and the Isoform Profile. Arthritis Rheumatol. 70, 255–265. 10.1002/art.40369 29108111

[B60] Hanson A. Brown M. A. (2017). Genetics and the Causes of Ankylosing Spondylitis. Rheum. Dis. Clin. North America 43, 401–414. 10.1016/j.rdc.2017.04.006 PMC6982368 28711142

[B61] Hao J. Liu Y. Xu J. Wang W. Wen Y. He A. (2017). Genome-wide DNA Methylation Profile Analysis Identifies Differentially Methylated Loci Associated with Ankylosis Spondylitis. Arthritis Res. Ther. 19, 177. 10.1186/s13075-017-1382-1 28743287 PMC5526246

[B62] He Z. Wang M. Li H. Wen C. (2019). GC-MS-based Fecal Metabolomics Reveals Gender-Attributed Fecal Signatures in Ankylosing Spondylitis. Sci. Rep. 9, 3872. 10.1038/s41598-019-40351-w 30846747 PMC6405849

[B63] Heinbockel T. Csoka A. (2018). Epigenetic Effects of Drugs of Abuse. Ijerph 15, 2098. 10.3390/ijerph15102098 PMC6210395 30257440

[B64] Hsu M.-T. Coca-Prados M. (1979). Electron Microscopic Evidence for the Circular Form of RNA in the Cytoplasm of Eukaryotic Cells. Nature 280, 339–340. 10.1038/280339a0 460409

[B65] Hu N. Chen X. Wang S. Yuan G. Wang Q. Shu H. (2021). The Association of Polymorphisms in TNF and Ankylosing Spondylitis in Common Population: a Meta-Analysis. Eur. Spine J. 30, 1402–1410. 10.1007/s00586-021-06845-w 33877454

[B66] Huang J. Song G. Yin Z. Luo X. Ye Z. (2014). Elevated miR-29a Expression Is Not Correlated with Disease Activity index in PBMCs of Patients with Ankylosing Spondylitis. Mod. Rheumatol. 24, 331–334. 10.3109/14397595.2013.854077 24593209

[B67] Huang J. Song G. Yin Z. Fu Z. Ye Z. (2017). MiR-29a and Messenger RNA Expression of Bone Turnover Markers in Canonical Wnt Pathway in Patients with Ankylosing Spondylitis. Clin. Lab. 63, 955–960. 10.7754/Clin.Lab.2017.161214 28627829

[B68] Huang J. Song G. Yin Z. Fu Z. Zhang L. (2019). Altered Expression of microRNAs Targeting DKK-1 in Peripheral Blood Mononuclear Cells of Patients with Ankylosing Spondylitis. cejoi 44, 59–64. 10.5114/ceji.2019.84018 PMC6526586 31114438

[B69] Huang R. Li F. Zhou Y. Zeng Z. He X. Fang L. (2020). Metagenome-wide Association Study of the Alterations in the Intestinal Microbiome Composition of Ankylosing Spondylitis Patients and the Effect of Traditional and Herbal Treatment. J. Med. Microbiol. 69, 797–805. 10.1099/jmm.0.001107 31778109 PMC7451032

[B70] Hwang M. C. Ridley L. Reveille J. D. (2021). Ankylosing Spondylitis Risk Factors: a Systematic Literature Review. Clin. Rheumatol. 40, 3079–3093. 10.1007/s10067-021-05679-7 33754220 PMC9044547

[B71] Ivanova M. Manolova I. Miteva L. Gancheva R. Stoilov R. Stanilova S. (2019). Genetic Variations in the *IL-12B* Gene in Association with IL-23 and IL-12p40 Serum Levels in Ankylosing Spondylitis. Rheumatol. Int. 39, 111–119. 10.1007/s00296-018-4204-0 30443744

[B72] Jah N. Jobart‐Malfait A. Ermoza K. Noteuil A. Chiocchia G. Breban M. (2020). HLA -B27 Subtypes Predisposing to Ankylosing Spondylitis Accumulate in an Endoplasmic Reticulum-Derived Compartment Apart from the Peptide‐Loading Complex. Arthritis Rheumatol. 72, 1534–1546. 10.1002/art.41281 32270915

[B73] Jamalyaria F. Ward M. M. Assassi S. Learch T. J. Lee M. Gensler L. S. (2017). Ethnicity and Disease Severity in Ankylosing Spondylitis a Cross-Sectional Analysis of Three Ethnic Groups. Clin. Rheumatol. 36, 2359–2364. 10.1007/s10067-017-3767-6 28780639 PMC5693696

[B74] Jandus C. Bioley G. Rivals J.-P. Dudler J. Speiser D. Romero P. (2008). Increased Numbers of Circulating Polyfunctional Th17 Memory Cells in Patients with Seronegative Spondylarthritides. Arthritis Rheum. 58, 2307–2317. 10.1002/art.23655 18668556

[B75] Jeanty C. Sourisce A. Noteuil A. Jah N. Wielgosik A. Fert I. (2014). HLA-B27 Subtype Oligomerization and Intracellular Accumulation Patterns Correlate with Predisposition to Spondyloarthritis. Arthritis Rheumatol. 66, 2113–2123. 10.1002/art.38644 24692163

[B76] Jiang Y. Ren Y. Zhou D. Xu Y. (2018). Associations between ERAP1 Polymorphisms and Susceptibility to Ankylosing Spondylitis. Medicine (Baltimore) 97, e13263. 10.1097/md.0000000000013263 30461632 PMC6393156

[B77] Jo S. Kang S. Han J. Choi S. H. Park Y.-S. Sung I.-H. (2018a). Accelerated Osteogenic Differentiation of Human Bone-Derived Cells in Ankylosing Spondylitis. J. Bone Miner. Metab. 36, 307–313. 10.1007/s00774-017-0846-3 28589411

[B78] Jo S. Wang S. E. Lee Y. L. Kang S. Lee B. Han J. (2018b). IL-17A Induces Osteoblast Differentiation by Activating JAK2/STAT3 in Ankylosing Spondylitis. Arthritis Res. Ther. 20, 115. 10.1186/s13075-018-1582-3 29880011 PMC5992730

[B79] Jo S. Han J. Lee Y. L. Yoon S. Lee J. Wang S. E. (2019). Regulation of Osteoblasts by Alkaline Phosphatase in Ankylosing Spondylitis. Int. J. Rheum. Dis. 22, 252–261. 10.1111/1756-185x.13419 30415492

[B80] Karami J. Mahmoudi M. Amirzargar A. Gharshasbi M. Jamshidi A. Aslani S. (2017). Promoter Hypermethylation of BCL11B Gene Correlates with Downregulation of Gene Transcription in Ankylosing Spondylitis Patients. Genes Immun. 18, 170–175. 10.1038/gene.2017.17 28794504

[B81] Kavadichanda C. G. Geng J. Bulusu S. N. Negi V. S. Raghavan M. (2021). Spondyloarthritis and the Human Leukocyte Antigen (HLA)-B*27 Connection. Front. Immunol. 12, 601518. 10.3389/fimmu.2021.601518 33763060 PMC7982681

[B82] Kenna T. J. Davidson S. I. Duan R. Bradbury L. A. McFarlane J. Smith M. (2012). Enrichment of Circulating Interleukin-17-Secreting Interleukin-23 Receptor-Positive γ/δ T Cells in Patients with Active Ankylosing Spondylitis. Arthritis Rheum. 64, 1420–1429. 10.1002/art.33507 22144400

[B83] Klingberg E. Magnusson M. K. Strid H. Deminger A. Ståhl A. Sundin J. (2019). A Distinct Gut Microbiota Composition in Patients with Ankylosing Spondylitis Is Associated with Increased Levels of Fecal Calprotectin. Arthritis Res. Ther. 21, 248. 10.1186/s13075-019-2018-4 31771630 PMC6880506

[B84] Kou J. Liu G. Liu X. Li T. Wei Y. Sun Y. (2020). Profiling and Bioinformatics Analysis of Differentially Expressed circRNAs in Spinal Ligament Tissues of Patients with Ankylosing Spondylitis. Biomed. Res. Int. 2020, 1–12. 10.1155/2020/7165893 PMC7313148 32626759

[B85] Lai N.-S. Yu H.-C. Chen H.-C. Yu C.-L. Huang H.-B. Lu M.-C. (2013). Aberrant Expression of microRNAs in T Cells from Patients with Ankylosing Spondylitis Contributes to the Immunopathogenesis. Clin. Exp. Immunol. 173, 47–57. 10.1111/cei.12089 23607629 PMC3694534

[B86] Lai N. L. Zhang S. X. Wang J. Zhang J. Q. Wang C. H. Gao C. (2019). The Proportion of Regulatory T Cells in Patients with Ankylosing Spondylitis: A Meta-Analysis. J. Immunol. Res. 2019, 1058738. 10.1155/2019/1058738 31772947 PMC6854227

[B87] Li M. Dai B. Tang Y. Lei L. Li N. Liu C. (2019a). Altered Bacterial-Fungal Interkingdom Networks in the Guts of Ankylosing Spondylitis Patients. mSystems 4, e00176–18. 10.1128/mSystems.00176-18 30944880 PMC6435815

[B88] Li X. Lv Q. Tu L. Zhao M. Zhang P. Li Q. (2019b). Aberrant Expression of microRNAs in Peripheral Blood Mononuclear Cells as Candidate Biomarkers in Patients with Axial Spondyloarthritis. Int. J. Rheum. Dis. 22, 1188–1195. 10.1111/1756-185x.13563 30990253

[B89] Li M. Zhou X. Zhou L. Yu Z. Fu L. Yang P. (2020a). Meta-analysis of Changes in the Number and Proportion of Regulatory T Cells in Patients with Ankylosing Spondylitis. Biomed. Res. Int. 2020, 1–15. 10.1155/2020/8709804 PMC7053470 32149142

[B90] Li Y. Zhang S. Zhang C. Wang M. (2020b). LncRNA MEG3 Inhibits the Inflammatory Response of Ankylosing Spondylitis by Targeting miR-146a. Mol. Cel. Biochem. 466, 17–24. 10.1007/s11010-019-03681-x 31894531

[B91] Liao H.-T. Lin Y.-F. Tsai C.-Y. Chou T.-C. (2018). Bone Morphogenetic Proteins and Dickkopf-1 in Ankylosing Spondylitis. Scand. J. Rheumatol. 47, 56–61. 10.1080/03009742.2017.1287305 28303752

[B92] Lim Kam Sian T. C. C. Indumathy S. Halim H. Greule A. Cryle M. J. Bowness P. (2019). Allelic Association with Ankylosing Spondylitis Fails to Correlate with Human Leukocyte Antigen B27 Homodimer Formation. J. Biol. Chem. 294, 20185–20195. 10.1074/jbc.ra119.010257 31740583 PMC6937573

[B93] Lin S. Qiu M. Chen J. (2015). IL-4 Modulates Macrophage Polarization in Ankylosing Spondylitis. Cell. Physiol. Biochem. 35, 2213–2222. 10.1159/000374026 25896783

[B94] Liu W. Wu Y. H. Zhang L. Liu X. Y. Xue B. Wang Y. (2015). Elevated Serum Levels of IL-6 and IL-17 May Associate with the Development of Ankylosing Spondylitis. Int. J. Clin. Exp. Med. 8, 17362–17376. 26770328 PMC4694228

[B95] Liu C.-H. Raj S. Chen C.-H. Hung K.-H. Chou C.-T. Chen I.-H. (2019a). HLA-B27-mediated Activation of TNAP Phosphatase Promotes Pathogenic Syndesmophyte Formation in Ankylosing Spondylitis. J. Clin. Invest. 129, 5357–5373. 10.1172/jci125212 31682238 PMC6877322

[B96] Liu W. Wang P. Xie Z. Wang S. Ma M. Li J. (2019b). Abnormal Inhibition of Osteoclastogenesis by Mesenchymal Stem Cells through the miR-4284/CXCL5 axis in Ankylosing Spondylitis. Cell Death Dis 10, 188. 10.1038/s41419-019-1448-x 30804325 PMC6389901

[B97] Liu Z. Huang F. Luo G. Wang Y. Du R. Sun W. (2020). miR-214 Stimulated by IL-17A Regulates Bone Loss in Patients with Ankylosing Spondylitis. Rheumatology 59, 1159–1169. 10.1093/rheumatology/kez594 31846044

[B36] López de Castro J. A. Alvarez-Navarro C. Brito A. Guasp P. Martín-Esteban A. Sanz-Bravo A. (2016). Molecular and Pathogenic Effects of Endoplasmic Reticulum Aminopeptidases ERAP1 and ERAP2 in MHC-I-Associated Inflammatory Disorders: Towards a Unifying View. Mol. Immunol. 77, 193–204. 10.1016/j.molimm.2016.08.005 27522479

[B99] Ma S. Wang D. D. Ma C. Y. Zhang Y. D. (2019). microRNA‐96 Promotes Osteoblast Differentiation and Bone Formation in Ankylosing Spondylitis Mice through Activating the Wnt Signaling Pathway by Binding to SOST. J. Cel Biochem. 120, 15429–15442. 10.1002/jcb.28810 31111563

[B100] Ma J. Zhang X. Zhang H. Chen H. (2020). lncRNA MEG3 Suppresses the Progression of Ankylosis Spondylitis by Regulating the Let-7i/SOST Axis. Front. Mol. Biosci. 7, 173. 10.3389/fmolb.2020.00173 32793634 PMC7393269

[B101] Madej M. Nowak B. Świerkot J. Sokolik R. Chlebicki A. Korman L. (2015). Cytokine Profiles in Axial Spondyloarthritis. Reumatologia. 1, 9–13. 10.5114/reum.2015.50551 PMC4847310 27407219

[B102] Martin E. M. Fry R. C. (2018). Environmental Influences on the Epigenome: Exposure- Associated DNA Methylation in Human Populations. Annu. Rev. Public Health 39, 309–333. 10.1146/annurev-publhealth-040617-014629 29328878

[B103] McGonagle D. Aydin S. Z. Gül A. Mahr A. Direskeneli H. (2015). 'MHC-I-opathy'-unified Concept for Spondyloarthritis and Behçet Disease. Nat. Rev. Rheumatol. 11, 731–740. 10.1038/nrrheum.2015.147 26526644

[B104] McGonagle D. Watad A. Sharif K. Bridgewood C. (2021). Why Inhibition of IL-23 Lacked Efficacy in Ankylosing Spondylitis. Front. Immunol. 12, 614255. 10.3389/fimmu.2021.614255 33815371 PMC8017223

[B105] Mei Y. Pan F. Gao J. Ge R. Duan Z. Zeng Z. (2011). Increased Serum IL-17 and IL-23 in the Patient with Ankylosing Spondylitis. Clin. Rheumatol. 30, 269–273. 10.1007/s10067-010-1647-4 21161669

[B106] Memczak S. Jens M. Elefsinioti A. Torti F. Krueger J. Rybak A. (2013). Circular RNAs Are a Large Class of Animal RNAs with Regulatory Potency. Nature 495, 333–338. 10.1038/nature11928 23446348

[B107] Meng S. Fan S. Li Y. Xu D. Ma X. Su Y. (2021). Aberrant Methylation of miR-34b and IL-12B mRNA Promoters Contributes to the Reduced Severity of Ankylosing Spondylitis. Biochem. Genet. 59, 714–730. 10.1007/s10528-020-10023-w 33512625

[B108] Michel M.-L. Pang D. J. Haque S. F. Y. Potocnik A. J. Pennington D. J. Hayday A. C. (2012). Interleukin 7 (IL-7) Selectively Promotes Mouse and Human IL-17-producing Cells. Proc. Natl. Acad. Sci. 109, 17549–17554. 10.1073/pnas.1204327109 23047700 PMC3491488

[B109] Milanez F. M. Saad C. G. S. VianaMaraes V. T. J. C. B. Moraes J. C. B. Périco G. V. Sampaio-Barros P. D. (2016). IL-23/Th17 axis Is Not Influenced by TNF-Blocking Agents in Ankylosing Spondylitis Patients. Arthritis Res. Ther. 18, 52. 10.1186/s13075-016-0949-6 26912133 PMC4765065

[B110] Miossec P. (2009). IL-17 and Th17 Cells in Human Inflammatory Diseases. Microbes Infect. 11, 625–630. 10.1016/j.micinf.2009.04.003 19371791

[B111] Mohammadi H. Hemmatzadeh M. Babaie F. Gowhari Shabgah A. Azizi G. Hosseini F. (2018). MicroRNA Implications in the Etiopathogenesis of Ankylosing Spondylitis. J. Cel. Physiol. 233, 5564–5573. 10.1002/jcp.26500 29377110

[B112] Motta F. Carena M. C. Selmi C. Vecellio M. (2020). MicroRNAs in Ankylosing Spondylitis: Function, Potential and Challenges. J. Translational Autoimmun. 3, 100050. 10.1016/j.jtauto.2020.100050 PMC7388379 32743531

[B113] Nakamura A. Boroojeni S. F. Haroon N. (2021). Aberrant Antigen Processing and Presentation: Key Pathogenic Factors Leading to Immune Activation in Ankylosing Spondylitis. Semin. Immunopathol. 43, 245–253. 10.1007/s00281-020-00833-w 33532928

[B114] Navid F. Holt V. Colbert R. A. (2021). The Enigmatic Role of HLA-B*27 in Spondyloarthritis Pathogenesis. Semin. Immunopathol 43, 235–243. 10.1007/s00281-021-00838-z 33481054

[B115] Ni W.-J. Leng X.-M. (2020). Down-regulated miR-495 Can Target Programmed Cell Death 10 in Ankylosing Spondylitis. Mol. Med. 26, 50. 10.1186/s10020-020-00157-3 32450789 PMC7249445

[B116] Page G. Miossec P. (2005). RANK and RANKL Expression as Markers of Dendritic Cell-T Cell Interactions in Paired Samples of Rheumatoid Synovium and Lymph Nodes. Arthritis Rheum. 52, 2307–2312. 10.1002/art.21211 16052586

[B117] Paladini F. Fiorillo M. T. Vitulano C. Tedeschi V. Piga M. Cauli A. (2018). An Allelic Variant in the Intergenic Region between ERAP1 and ERAP2 Correlates with an Inverse Expression of the Two Genes. Sci. Rep. 8, 10398. 10.1038/s41598-018-28799-8 29991817 PMC6039459

[B118] Paladini F. Fiorillo M. T. Tedeschi V. D’Otolo V. Piga M. Cauli A. (2019). The Rs75862629 Minor Allele in the Endoplasmic Reticulum Aminopeptidases Intergenic Region Affects Human Leucocyte Antigen B27 Expression and Protects from Ankylosing Spondylitis in Sardinia. Rheumatology 58, 2315–2324. 10.1093/rheumatology/kez212 31209470

[B119] Park N. J. Zhou H. Elashoff D. Henson B. S. Kastratovic D. A. Abemayor E. (2009). Salivary microRNA: Discovery, Characterization, and Clinical Utility for Oral Cancer Detection. Clin. Cancer Res. 15, 5473–5477. 10.1158/1078-0432.ccr-09-0736 19706812 PMC2752355

[B120] Pauley K. M. Cha S. Chan E. K. L. (2009). MicroRNA in Autoimmunity and Autoimmune Diseases. J. Autoimmun. 32, 189–194. 10.1016/j.jaut.2009.02.012 19303254 PMC2717629

[B121] Pepelyayeva Y. Rastall D. P. W. Aldhamen Y. A. O’Connell P. Raehtz S. Alyaqoub F. S. (2018). ERAP1 Deficient Mice Have Reduced Type 1 Regulatory T Cells and Develop Skeletal and Intestinal Features of Ankylosing Spondylitis. Sci. Rep. 8, 12464. 10.1038/s41598-018-30159-5 30127455 PMC6102283

[B122] Perez-Sanchez C. Font-Ugalde P. Ruiz-Limon P. Lopez-Pedrera C. Castro-Villegas M. C. Abalos-Aguilera M. C. (2018). Circulating microRNAs as Potential Biomarkers of Disease Activity and Structural Damage in Ankylosing Spondylitis Patients. Hum. Mol. Genet. 27, 875–890. 10.1093/hmg/ddy008 29329380

[B123] Prajzlerová K. Grobelná K. Hušáková M. Forejtová S. Jüngel A. Gay S. (2017). Association between Circulating miRNAs and Spinal Involvement in Patients with Axial Spondyloarthritis. PLos One 12, e185323. 10.1371/journal.pone.0185323 PMC5609864 28938006

[B124] Qian B.-P. Ji M.-L. Qiu Y. Wang B. Yu Y. Shi W. (2016). Identification of Serum miR-146a and miR-155 as Novel Noninvasive Complementary Biomarkers for Ankylosing Spondylitis. Spine 41, 735–742. 10.1097/brs.0000000000001339 27128253

[B125] Qin X. Zhu B. Jiang T. Tan J. Wu Z. Yuan Z. (2019). miR-17-5p Regulates Heterotopic Ossification by Targeting ANKH in Ankylosing Spondylitis. Mol. Ther. - Nucleic Acids 18, 696–707. 10.1016/j.omtn.2019.10.003 31726387 PMC6859287

[B126] Qu S. Yang X. Li X. Wang J. Gao Y. Shang R. (2015). Circular RNA: A New star of Noncoding RNAs. Cancer Lett. 365, 141–148. 10.1016/j.canlet.2015.06.003 26052092

[B127] Redeker I. Siegmund B. Ghoreschi K. Pleyer U. Callhoff J. Hoffmann F. (2020). The Impact of Extra-musculoskeletal Manifestations on Disease Activity, Functional Status, and Treatment Patterns in Patients with Axial Spondyloarthritis: Results from a Nationwide Population-Based Study. Ther. Adv. Musculoskelet. Dis. 12, 1759720X20972610. 10.1177/1759720X20972610 PMC7682214 33281952

[B128] Regner E. H. Ohri N. Stahly A. Gerich M. E. Fennimore B. P. Ir D. (2018). Functional Intraepithelial Lymphocyte Changes in Inflammatory Bowel Disease and Spondyloarthritis Have Disease Specific Correlations with Intestinal Microbiota. Arthritis Res. Ther. 20, 149. 10.1186/s13075-018-1639-3 30029674 PMC6053728

[B129] Reinhardt A. Yevsa T. Worbs T. Lienenklaus S. Sandrock I. Oberdörfer L. (2016). Interleukin-23-Dependent γ/δ T Cells Produce Interleukin-17 and Accumulate in the Enthesis, Aortic Valve, and Ciliary Body in Mice. Arthritis Rheumatol. 68, 2476–2486. 10.1002/art.39732 27111864

[B130] Reveille J. D. Zhou X. LeeWeisman M. M. H. Weisman M. H. Yi L. Gensler L. S. (2019). HLA Class I and II Alleles in Susceptibility to Ankylosing Spondylitis. Ann. Rheum. Dis. 78, 66–73. 10.1136/annrheumdis-2018-213779 30341055 PMC6982366

[B131] Reveille J. D. (2012). Genetics of Spondyloarthritis-Beyond the MHC. Nat. Rev. Rheumatol. 8, 296–304. 10.1038/nrrheum.2012.41 22487796

[B132] Reyes-Loyola P. Rodríguez-Henríquez P. Ballinas-Verdugo M. A. Amezcua-Castillo L. M. Juárez-Vicuña Y. Jiménez-Rojas V. (2019). Plasma Let-7i, miR-16, and miR-221 Levels as Candidate Biomarkers for the Assessment of Ankylosing Spondylitis in Mexican Patients Naïve to Anti-TNF Therapy. Clin. Rheumatol. 38, 1367–1373. 10.1007/s10067-019-04509-1 30911942

[B133] Rezaiemanesh A. Mahmoudi M. Amirzargar A. A. Vojdanian M. Jamshidi A. R. Nicknam M. H. (2017). Ankylosing Spondylitis M-CSF-Derived Macrophages Are Undergoing Unfolded Protein Response (UPR) and Express Higher Levels of Interleukin-23. Mod. Rheumatol. 27, 862–867. 10.1080/14397595.2016.1259716 27846758

[B134] Rezaiemanesh A. Abdolmaleki M. Abdolmohammadi K. Aghaei H. Pakdel F. D. Fatahi Y. (2018). Immune Cells Involved in the Pathogenesis of Ankylosing Spondylitis. Biomed. Pharmacother. 100, 198–204. 10.1016/j.biopha.2018.01.108 29428668

[B135] Rihl M. Kellner H. Kellner W. Barthel C. Yu D. T. Y. Tak P. P. (2008). Identification of Interleukin-7 as a Candidate Disease Mediator in Spondylarthritis. Arthritis Rheum. 58, 3430–3435. 10.1002/art.23998 18975340

[B136] Robinson P. C. Costello M. E. Leo P. Bradbury L. A. Hollis K. Cortes A. (2015). ERAP2 Is Associated with Ankylosing Spondylitis in HLA-B27-Positive and HLA-B27-Negative Patients. Ann. Rheum. Dis. 74, 1627–1629. 10.1136/annrheumdis-2015-207416 25917849 PMC4498996

[B137] Rosenbaum J. T. Asquith M. (2018). The Microbiome and HLA-B27-Associated Acute Anterior Uveitis. Nat. Rev. Rheumatol. 14, 704–713. 10.1038/s41584-018-0097-2 30301938 PMC6597169

[B138] Russell T. Bridgewood C. Rowe H. Altaie A. Jones E. McGonagle D. (2021). Cytokine "fine Tuning" of Enthesis Tissue Homeostasis as a Pointer to Spondyloarthritis Pathogenesis with a Focus on Relevant TNF and IL-17 Targeted Therapies. Semin. Immunopathol 43, 193–206. 10.1007/s00281-021-00836-1 33544244 PMC7990848

[B139] Sahlberg A. S. Ruuska M. Colbert R. A. Granfors K. Penttinen M. A. (2012). Altered PKR Signalling and C/EBPβ Expression Is Associated with HLA-B27 Expression in Monocytic Cells. Scand. J. Immunol. 75, 184–192. 10.1111/j.1365-3083.2011.02648.x 21988375 PMC3271165

[B140] SanterBar L. C. Bär C. Thum T. (2019). Circular RNAs: A Novel Class of Functional RNA Molecules with a Therapeutic Perspective. Mol. Ther. 27, 1350–1363. 10.1016/j.ymthe.2019.07.001 31324392 PMC6697450

[B141] Shabgah A. G. Navashenaq J. G. Shabgah O. G. Mohammadi H. Sahebkar A. (2017). Interleukin-22 in Human Inflammatory Diseases and Viral Infections. Autoimmun. Rev. 16, 1209–1218. 10.1016/j.autrev.2017.10.004 29037907

[B142] Sharif K. Bridgewood C. Dubash S. McGonagle D. (2020). Intestinal and Enthesis Innate Immunity in Early Axial Spondyloarthropathy. Rheumatology (Oxford) 59 (Suppl. l), iv67–iv78. 10.1093/rheumatology/keaa408 33053197 PMC7566539

[B143] Sharip A. Kunz J. (2020). Understanding the Pathogenesis of Spondyloarthritis. Biomolecules 10, 1461. 10.3390/biom10101461 PMC7588965 33092023

[B144] Shen H. Goodall J. C. Hill Gaston J. S. (2009). Frequency and Phenotype of Peripheral Blood Th17 Cells in Ankylosing Spondylitis and Rheumatoid Arthritis. Arthritis Rheum. 60, 1647–1656. 10.1002/art.24568 19479869

[B145] Sherlock J. P. Joyce-Shaikh B. Turner S. P. Chao C.-C. Sathe M. Grein J. (2012). IL-23 Induces Spondyloarthropathy by Acting on ROR-Γt+ CD3+CD4−CD8− Entheseal Resident T Cells. Nat. Med. 18, 1069–1076. 10.1038/nm.2817 22772566

[B146] Shiue I. (2015). Relationship of Environmental Exposures and Ankylosing Spondylitis and Spinal Mobility: US NHAENS, 2009-2010. Int. J. Environ. Health Res. 25, 322–329. 10.1080/09603123.2014.945512 25103950

[B147] Singh A. K. Misra R. Aggarwal A. (2011). Th-17 Associated Cytokines in Patients with Reactive Arthritis/undifferentiated Spondyloarthropathy. Clin. Rheumatol. 30, 771–776. 10.1007/s10067-010-1646-5 21181220

[B148] Slobodin G. Kessel A. Kofman N. Toubi E. Rosner I. Odeh M. (2012). Phenotype of Resting and Activated Monocyte-Derived Dendritic Cells Grown from Peripheral Blood of Patients with Ankylosing Spondylitis. Inflammation 35, 772–775. 10.1007/s10753-011-9373-x 21833763

[B149] Stoll M. L. Kumar R. Morrow C. D. Lefkowitz E. J. Cui X. Genin A. (2014). Altered Microbiota Associated with Abnormal Humoral Immune Responses to Commensal Organisms in Enthesitis-Related Arthritis. Arthritis Res. Ther. 16, 486. 10.1186/s13075-014-0486-0 25434931 PMC4272554

[B150] Stolwijk C. van Tubergen A. Castillo-Ortiz J. D. Boonen A. (2015). Prevalence of Extra-articular Manifestations in Patients with Ankylosing Spondylitis: a Systematic Review and Meta-Analysis. Ann. Rheum. Dis. 74, 65–73. 10.1136/annrheumdis-2013-203582 23999006

[B151] Sutherland G. R. Baker E. Fernandez K. E. Callen D. F. Goodwin R. G. Lupton S. (1989). The Gene for Human Interleukin 7 (*IL7*) Is at 8q12-13. Hum. Genet. 82, 371–372. 10.1007/BF00274000 2786840

[B152] Sveaas S. Berg I. Provan S. Semb A. Olsen I. Ueland T. (2015). Circulating Levels of Inflammatory Cytokines and Cytokine Receptors in Patients with Ankylosing Spondylitis: a Cross-Sectional Comparative Study. Scand. J. Rheumatol. 44, 118–124. 10.3109/03009742.2014.956142 25756521

[B153] Tabrizi Z. Mansouri R. Aslani S. Jamshidi A. R. Mahmoudi M. (2017). Expression Levels of the microRNA Maturing Microprocessor Complex Components; Drosha, Dicer, and DGCR8 in PBMCs from Ankylosing Spondylitis Patients. Mjr 28, 80–85. 10.31138/mjr.28.2.80 32185262 PMC7046025

[B154] Talpin A. Costantino F. Bonilla N. Leboime A. Letourneur F. Jacques S. (2014). Monocyte-derived Dendritic Cells from HLA-B27+ Axial Spondyloarthritis (SpA) Patients Display Altered Functional Capacity and Deregulated Gene Expression. Arthritis Res. Ther. 16, 417. 10.1186/s13075-014-0417-0 25142923 PMC4292999

[B155] Tang X.-Z. Jo J. Tan A. T. Sandalova E. Chia A. Tan K. C. (2013). IL-7 Licenses Activation of Human Liver Intrasinusoidal Mucosal-Associated Invariant T Cells. J.I. 190, 3142–3152. 10.4049/jimmunol.1203218 23447689

[B156] Tang S. L. Huang Q. H. Wu L. G. Liu C. Cai A. L. (2018). MiR-124 Regulates Osteoblast Differentiation through GSK-3β in Ankylosing Spondylitis. Eur. Rev. Med. Pharmacol. Sci. 22, 6616–6624. 10.26355/eurrev_201810_16136 30402833

[B157] Tito R. Y. Cypers H. Joossens M. Varkas G. Van PraetGlorieus L. E. Glorieus E. (2017). Brief Report: *Dialister* as a Microbial Marker of Disease Activity in Spondyloarthritis. Arthritis Rheumatol. 69, 114–121. 10.1002/art.39802 27390077

[B158] Toussirot E. Abbas W. KhanTissot K. A. M. Tissot M. Jeudy A. Baud L. (2013). Imbalance between HAT and HDAC Activities in the PBMCs of Patients with Ankylosing Spondylitis or Rheumatoid Arthritis and Influence of HDAC Inhibitors on TNF Alpha Production. PLoS One 8, e70939. 10.1371/journal.pone.0070939 24039666 PMC3748901

[B159] Venken K. Elewaut D. (2015). IL-23 Responsive Innate-like T Cells in Spondyloarthritis: the Less Frequent They Are, the More Vital They Appear. Curr. Rheumatol. Rep. 17, 30. 10.1007/s11926-015-0507-2 25874346

[B160] Vitulano C. Tedeschi V. Paladini F. Sorrentino R. Fiorillo M. T. (2017). The Interplay between HLA-B27 and ERAP1/ERAP2 Aminopeptidases: from Anti-viral protection to Spondyloarthritis. Clin. Exp. Immun. 190, 281–290. 10.1111/cei.13020 28759104 PMC5680067

[B161] Voruganti A. Bowness P. (2020). New Developments in Our Understanding of Ankylosing Spondylitis Pathogenesis. Immunology 161, 94–102. 10.1111/imm.13242 32696457 PMC7496782

[B162] Wakkach A. Fournier N. Brun V. Breittmayer J.-P. Cottrez F. Groux H. (2003). Characterization of Dendritic Cells that Induce Tolerance and T Regulatory 1 Cell Differentiation *In Vivo* . Immunity 18, 605–617. 10.1016/s1074-7613(03)00113-4 12753738

[B163] Wang X. Guo B. Li Q. Peng J. Yang Z. Wang A. (2013). miR-214 Targets *ATF4* to Inhibit Bone Formation. Nat. Med. 19, 93–100. 10.1038/nm.3026 23223004

[B164] Wang M. Ji B. Wang J. Cheng X. Zhou Q. Zhou J. (2014). Tim-3 Polymorphism Downregulates Gene Expression and Is Involved in the Susceptibility to Ankylosing Spondylitis. DNA Cel Biol. 33, 723–728. 10.1089/dna.2014.2456 24905803

[B165] Wang J. Zhao Q. Wang G. Yang C. Xu Y. Li Y. (2016). Circulating Levels of Th1 and Th2 Chemokines in Patients with Ankylosing Spondylitis. Cytokine 81, 10–14. 10.1016/j.cyto.2016.01.012 26827189

[B166] Wang M. Liu C. Bond A. Yang J. Zhou X. Wang J. (2018). Dysfunction of Regulatory T Cells in Patients with Ankylosing Spondylitis Is Associated with a Loss of Tim-3. Int. Immunopharmacology 59, 53–60. 10.1016/j.intimp.2018.03.032 29625390

[B167] Wang Y. Liu J. Ma J. Sun T. Zhou Q. Wang W. (2019). Exosomal circRNAs: Biogenesis, Effect and Application in Human Diseases. Mol. Cancer 18, 116. 10.1186/s12943-019-1041-z 31277663 PMC6610963

[B168] Watad A. Cuthbert R. J. Amital H. McGonagle D. (2018). Enthesitis: Much More Than Focal Insertion point Inflammation. Curr. Rheumatol. Rep. 20, 41. 10.1007/s11926-018-0751-3 29846815 PMC5976708

[B169] Watad A. Rowe H. Russell T. Zhou Q. Anderson L. K. Khan A. (2020). Normal Human Enthesis Harbours Conventional CD4+ and CD8+ T Cells with Regulatory Features and Inducible IL-17A and TNF Expression. Ann. Rheum. Dis. 79, 1044–1054. 10.1136/annrheumdis-2020-217309 32404344 PMC7392498

[B170] Wen C. Zheng Z. Shao T. Liu L. Xie Z. Le Chatelier E. (2017). Quantitative Metagenomics Reveals Unique Gut Microbiome Biomarkers in Ankylosing Spondylitis. Genome Biol. 18, 142. 10.1186/s13059-017-1271-6 28750650 PMC5530561

[B171] Wendling D. Cedoz J.-P. Racadot E. Dumoulin G. (2007). Serum IL-17, BMP-7, and Bone Turnover Markers in Patients with Ankylosing Spondylitis. Jt. Bone Spine 74, 304–305. 10.1016/j.jbspin.2006.11.005 17369068

[B172] Whyte J. M. Ellis J. J. Brown M. A. Kenna T. J. (2019). Best Practices in DNA Methylation: Lessons from Inflammatory Bowel Disease, Psoriasis and Ankylosing Spondylitis. Arthritis Res. Ther. 21, 133. 10.1186/s13075-019-1922-y 31159831 PMC6547594

[B173] Wordsworth B. P. Cohen C. J. Davidson C. Vecellio M. (2021). Perspectives on the Genetic Associations of Ankylosing Spondylitis. Front. Immunol. 12, 603726. 10.3389/fimmu.2021.603726 33746951 PMC7977288

[B174] Wright P. B. McEntegart A. McCarey D. McInnes I. B. Siebert S. Milling S. W. F. (2016). Ankylosing Spondylitis Patients Display Altered Dendritic Cell and T Cell Populations that Implicate Pathogenic Roles for the IL-23 Cytokine axis and Intestinal Inflammation. Rheumatology 55, 120–132. 10.1093/rheumatology/kev245 26320138 PMC4676904

[B175] Wu Y. Ren M. Yang R. Liang X. Ma Y. Tang Y. (2011). Reduced Immunomodulation Potential of Bone Marrow-Derived Mesenchymal Stem Cells Induced CCR4+CCR6+Th/Treg Cell Subset Imbalance in Ankylosing Spondylitis. Arthritis Res. Ther. 13, R29. 10.1186/ar3257 21338515 PMC3241373

[B176] Xia Y. Liang Y. Guo S. Yu J.-G. Tang M.-S. Xu P.-H. (2018). Association between Cytokine Gene Polymorphisms and Ankylosing Spondylitis Susceptibility: a Systematic Review and Meta-Analysis. Postgrad. Med. J. 94, 508–516. 10.1136/postgradmedj-2018-135665 30322951

[B177] Xu H. Yin J. (2019). HLA Risk Alleles and Gut Microbiome in Ankylosing Spondylitis and Rheumatoid Arthritis. Best Pract. Res. Clin. Rheumatol. 33, 101499. 10.1016/j.berh.2020.101499 32279929

[B178] Xueyi L. Lina C. Zhenbiao W. Qing H. Qiang L. Zhu P. (2013). Levels of Circulating Th17 Cells and Regulatory T Cells in Ankylosing Spondylitis Patients with an Inadequate Response to Anti−TNF-α Therapy. J. Clin. Immunol. 33, 151–161. 10.1007/s10875-012-9774-0 22926407

[B98] Yang K. L. Lejeune A. Chang G. Scher J. U. Koralov S. B. (2021). Microbial-derived Antigens and Metabolites in Spondyloarthritis. Semin. Immunopathol. 43, 163–172. 10.1007/s00281-021-00844-1 33569635

[B179] Yang P. T. Kasai H. Zhao L. J. Xiao W. G. Tanabe F. Ito M. (2004). Increased CCR4 Expression on Circulating CD4+ T Cells in Ankylosing Spondylitis, Rheumatoid Arthritis and Systemic Lupus Erythematosus. Clin. Exp. Immunol. 138, 342–347. 10.1111/j.1365-2249.2004.02617.x 15498047 PMC1809206

[B180] Yang L. Wang L. Wang X. Xian C. Lu H. (2016). A Possible Role of Intestinal Microbiota in the Pathogenesis of Ankylosing Spondylitis. Ijms 17, 2126. 10.3390/ijms17122126 PMC5187926 27999312

[B181] Yang W. Yan X. Xia Q. Tao Q. Gan X. Zhang Y. (2019). Predisposition of Six Well-Characterized microRNAs to Syndesmophytes Among Chinese Patients with Ankylosing Spondylitis. Mod. Rheumatol. 29, 173–180. 10.1080/14397595.2018.1453277 29542383

[B182] Zeboulon-Ktorza N. Boelle P. Y. Nahal R. S. D'agostino M. A. Vibert J. F. Turbelin C. (2013). Influence of Environmental Factors on Disease Activity in Spondyloarthritis: a Prospective Cohort Study. J. Rheumatol. 40, 469–475. 10.3899/jrheum.121081 23418385

[B183] Zeng L. Lindstrom M. J. Smith J. A. (2011). Ankylosing Spondylitis Macrophage Production of Higher Levels of Interleukin-23 in Response to Lipopolysaccharide without Induction of a Significant Unfolded Protein Response. Arthritis Rheum. 63, 3807–3817. 10.1002/art.30593 22127699 PMC3228355

[B184] Zhang L. Li Y.-G. Li Y.-H. Qi L. Liu X.-G. Yuan C.-Z. (2012). Increased Frequencies of Th22 Cells as Well as Th17 Cells in the Peripheral Blood of Patients with Ankylosing Spondylitis and Rheumatoid Arthritis. PLoS One 7, e31000. 10.1371/journal.pone.0031000 22485125 PMC3317658

[B185] Zhang C. Wang C. Jia Z. Tong W. Liu D. He C. (2017). Differentially Expressed mRNAs, lncRNAs, and miRNAs with Associated Co-expression and ceRNA Networks in Ankylosing Spondylitis. Oncotarget 8, 113543–113557. 10.18632/oncotarget.22708 29371928 PMC5768345

[B186] Zhang X. Lu J. Pan Z. Ma Y. Liu R. Yang S. (2019). DNA Methylation and Transcriptome Signature of the IL12B Gene in Ankylosing Spondylitis. Int. Immunopharmacology 71, 109–114. 10.1016/j.intimp.2019.03.026 30889422

[B187] Zhao S.-S. Hu J.-W. Wang J. Lou X.-J. Zhou L.-L. (2011). Inverse Correlation between CD4+CD25HighCD127low/− Regulatory T-Cells and Serum Immunoglobulin A in Patients with New-Onset Ankylosing Spondylitis. J. Int. Med. Res. 39, 1968–1974. 10.1177/147323001103900543 22118001

[B188] Zhao C. Sun W. Zhang P. Ling S. Li Y. Zhao D. (2015). miR-214 Promotes Osteoclastogenesis by Targeting Pten/PI3k/Akt Pathway. RNA Biol. 12, 343–353. 10.1080/15476286.2015.1017205 25826666 PMC4615895

[B189] Zhao J. Zhang Y. Liu B. (2020). MicroRNA-204-5p I-nhibits the O-steogenic D-ifferentiation of A-nkylosing S-pondylitis F-ibroblasts by R-egulating the Notch2 S-ignaling P-athway. Mol. Med. Rep. 22, 2537–2544. 10.3892/mmr.2020.11303 32705191 PMC7411397

[B190] Zhou L. Xu H. Hu L. Xie Y. Lu H. Zhang Z. (2015). Decreased Programmed Death-1 Expression on the T Cells of Patients with Ankylosing Spondylitis. Am. J. Med. Sci. 349, 488–492. 10.1097/maj.0000000000000468 25881983

